# IRFinder: assessing the impact of intron retention on mammalian gene expression

**DOI:** 10.1186/s13059-017-1184-4

**Published:** 2017-03-15

**Authors:** Robert Middleton, Dadi Gao, Aubin Thomas, Babita Singh, Amy Au, Justin J-L Wong, Alexandra Bomane, Bertrand Cosson, Eduardo Eyras, John E. J. Rasko, William Ritchie

**Affiliations:** 1 Bioinformatics Laboratory, Centenary Institute, Camperdown, 2050 Australia; 2 Molecular Neurogenetics Unit, Center for Human Genetic Research, Massachusetts General Hospital, Boston, MA USA; 3 Boston & Harvard Medical School, Boston, MA USA; 4 Gene & Stem Cell Therapy Program, Centenary Institute, Camperdown, 2050 Australia; 5 CNRS, UPR 1142, Montpellier, 34094 France; 6 Pompeu Fabra University, UPF, Dr. Aiguader 88, E08003 Barcelona, Spain; 7 Sydney Medical School, University of Sydney, Sydney, NSW 2006 Australia; 8 Gene Regulation in Cancer Laboratory, Centenary Institute, University of Sydney, Camperdown, 2050 Australia; 9 Université Paris Diderot, Sorbonne Paris Cité, Epigenetics and Cell Fate, UMR7216, CNRS, F-75013 Paris, France; 10 Catalan Institution for Research and Advanced Studies, ICREA, Passeig Lluís Companys 23, E08010 Barcelona, Spain; 11 Cell and Molecular Therapies, Royal Prince Alfred Hospital, Camperdown, 2050 Australia; 12 CNRS, UMR 5203, Montpellier, 34094 France

**Keywords:** mRNA splicing, Intron retention, Gene regulation

## Abstract

Intron retention (IR) occurs when an intron is transcribed into pre-mRNA and remains in the final mRNA. We have developed a program and database called IRFinder to accurately detect IR from mRNA sequencing data. Analysis of 2573 samples showed that IR occurs in all tissues analyzed, affects over 80% of all coding genes and is associated with cell differentiation and the cell cycle. Frequently retained introns are enriched for specific RNA binding protein sites and are often retained in clusters in the same gene. IR is associated with lower protein levels and intron-retaining transcripts that escape nonsense-mediated decay are not actively translated.

**Electronic supplementary material:**

The online version of this article (doi:10.1186/s13059-017-1184-4) contains supplementary material, which is available to authorized users.

## Background

Alternative splicing (AS) affects up to 95% of multi-exonic genes in humans [1]. The three main types of AS are exon skipping, alternative 5′ or 3′ usage and intron retention (IR). IR occurs when an intron is transcribed into pre-mRNA and remains in the final mRNA. It constitutes a class of AS that is often neglected because these events are difficult to measure reliably. IR can introduce functional elements within mRNAs [2] or alternatively may lead to the introduction of premature termination codons, resulting in degradation of the mRNA by a surveillance mechanism called nonsense-mediated decay (NMD) [3]. This process of IR followed by NMD can downregulate up to 35% of alternatively spliced transcripts in mammals [4]. The NMD pathway is absolutely essential for post-implantation embryonic development as shown by Upf1 nullizygosity [5]. However, an obvious consequence of NMD is that mRNAs containing introns with premature termination codons are degraded and therefore difficult to quantify. Consequently, the role of IR in specific eukaryotic biological pathways has been poorly defined prior to the availability of ultra-deep sequencing technologies.

We recently discovered that IR combined with the NMD pathway is not a by-product of faulty splicing but rather a major driver of the cellular differentiation of granulocytes [6]. In this pioneering study we developed an approach to correctly identify introns that are differentially retained during granulocytic differentiation from sequencing data. Our approach was subsequently used to uncover the role of IR in stem cell reprogramming where it regulated demethylation genes at specific stages of reprogramming [7]. Since then, other publications have highlighted the importance of IR in gene regulation [8], differentiation [9], and cancer [10]. Despite increasing evidence that IR can regulate hundreds of genes in numerous systems, current studies still fail to identify IR events in their transcriptomic data.

Here we have developed a significantly enhanced program in terms of sensitivity and speed for detecting retained introns and filtering samples that are inappropriate for IR analysis in terms of library preparation and quality. This novel program was validated using quantitative RT-PCR (RT-qPCR) against retained introns and a NMD knockdown experiment. IRFinder correctly identified IR events and measured the ratio of retained introns to correctly spliced introns with great accuracy. Using IRFinder, we analyzed 3435 human samples, of which 2573 were suitable for analysis. We found that IR occurs in hundreds of genes in all tissues analyzed, affects over 80% of all coding genes, and is associated with cell differentiation and cell cycle. Retained introns in the same genes are frequently adjacent to each other and intron-retaining genes cluster closely on the genome, suggesting a global mechanism that regulates multiple introns simultaneously. By analyzing mass spectrometry and ribosome sequencing data we discovered that intron-retaining genes have lower protein output and that IR transcripts that escape NMD are not actively translated. Finally, by comparing introns that are frequently retained amongst the 2573 samples with introns that are rarely retained, we discovered a distinct primary sequence signature amongst frequently retained introns that is enriched in RNA binding protein sites. These proteins modulate the level of IR and thus the level of repression of frequently retained introns. Our program to analyze IR from sequencing data is available at GitHub (https://github.com/williamritchie/IRFinder) and a database of IR in over 2000 human samples is freely available at IRBase (http://mimirna.centenary.org.au/irfinder/database/).

### Implementation

Fair measurement of intronic expression is challenged by numerous factors. Within introns, highly expressed features such as snoRNAs, microRNAs, or unannotated exons may erroneously inflate count-based measures of intronic expression. Conversely, low complexity regions, common in introns, prevent unique mapping of reads. Because retained introns are generally expressed at a fraction of their flanking exons, uncorrected biases can massively disrupt IR estimation.

IRFinder implements an end-to-end analysis of retained introns from mRNA sequencing data in multiple species. It includes alignment via the STAR algorithm, quality controls on the sample analyzed, IR detection, and quantification and statistics for comparing IR levels between multiple samples. We provide standalone scripts for each of these steps so they can be used independently and provide command line tools to chain them together for complete analysis. On pre-aligned sequencing data, our program can run on a desktop computer with less than 2 GB of memory and takes approximately 10 minutes to detect IR events. Because we use STAR to align reads, our end-to-end analysis with raw reads requires at least 48 GB of memory and depends mainly on STAR runtime.

Our tool facilitates the analysis of large amounts of online data by automatically testing samples for their suitability for IR detection. Unsuitable samples either have high levels of DNA contamination or have been mislabeled as mRNA sequencing when in fact they are other types of experiments such as genome sequencing or ChIP-seq. Although this information should be available from online repositories, we found that only 68% (2573/3774) were suitable for analysis in this study. This was mainly due to the incorrect use of the term “mRNA-Seq”, which was frequently used in whole RNA experiments and CHIP-Seq experiments. Our approach also uses a series of programmatically fast steps written in C++ that automatically detect and trim adapters from sequencing reads. Of the 3435 initial samples we analyzed, 3096 (91%) still had adapters in the sequencing reads despite having been already processed by an adaptor-trimming algorithm.

IRFinder was capable of estimating IR events with low coverage or low mappability as confirmed by RT-qPCR (Additional file 1: Figure S1a–e). When compared with the currently available tools MISO and DEXseq, our IRFinder had higher accuracy and precision (Additional file 2: Text, Figure S2, Tables S2 and S3).

The IRFinder algorithm and instruction manual are available at GitHub (https://github.com/williamritchie/IRFinder).

## Results

### IRFinder detects IR in over 80% of coding genes and in all samples tested

**Fig. 1:**
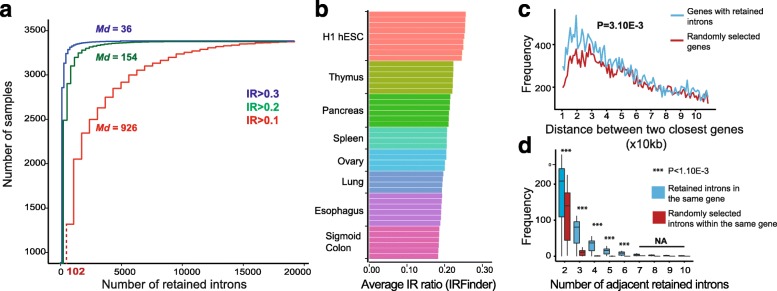
Intron retention is widespread and its measurement is consistent within the same tissue type. **a** Number of retained introns in each of the 2573 downloaded samples that were retained in more than 10% (*red*), 20% (*green*), 30% (*blue*) of transcripts (IR ratio >0.1, 0.2, 0.3). **b** Average IR ratio in eight different tissue types. *hESC* human embryonic stem cell. **c** Genes with IR events occur closer than expected. Genomic distance between genes with IR events (*blue*) compared to a control of the same number of randomly selected expressed genes. **d** Retained introns within the same gene are often adjacent to each other. Number of introns that are separated by only one exon (*x-axis*) in the same gene (*blue*) compared to a control where the number of IR events within each gene are randomly distributed amongst introns

To demonstrate the functionality of our method, we downloaded 3774 human samples from the European Nucleotide Archive (http://www.ebi.ac.uk/ena). These were annotated as mRNA-Seq experiments in the archive with over 50 million reads each. The IRFinder quality control filter (see “Methods”) labeled 1201 of these as unfit for further analysis because they were either not RNA-seq experiments or not enriched for poly(A) tailed mRNAs. We searched for retained introns in 2573 remaining samples. We focused on introns that were retained in more than 10% of transcripts (IR ratio >0.1) with at least a coverage of three reads across the entire intron after excluding non-measurable intronic regions (see “Methods”). We found that 87.9% (16,307/18,560) of all multi-exonic protein coding genes with sufficient coverage had retained introns in at least three samples in our dataset. None of these 16,307 was retained in all of the samples and the majority (95%) of introns were retained in less than 7% of samples, suggesting that the IR events we detected were specific to tissue or cell types. All samples had over 100 retained introns with a median of 926 retained introns (Fig. 1a) per sample. This means that IR is more widespread than previously expected with 87.9% of protein coding genes and all tissue types showing high levels of IR.

To determine how consistent IR levels were within the same tissue type we calculated the average IR ratio of retained introns in eight distinct tissue types for which we had over three replicates. We found that there was a significant overlap in genes for which we detected retained introns between each replicate of the same tissue (*p* < 2.10E-6, hypergeometric test) and found that the average IR ratio was homogenous within each tissue (Fig. 1b).

Having observed that numerous genes had multiple retained introns and that these were often adjacent to each other (separated by only one exon), we calculated the distance between genes with retained introns (Fig. 1c) and how often retained introns within the same gene were adjacent to each other (Fig. 1d). We found that adjacent introns within the same gene were more frequently retained than expected and that genes with retained introns were significantly closer to each other than the same number of randomly selected expressed genes (Mann–Whitney test *p* = 3.10-3; Additional file 3). Although in this study we were able to confirm that retained introns were generally surrounded by weaker splice sites [8] (Additional file 4: Text and Figure S3), the above results on IR clusters indicate that IR is not solely regulated at each splicing junction but is also regulated by a more global means of regulation that encompasses multiple introns and even multiple genes. Multiple retained introns within the same gene could be regulated by transcription rate through the gene in agreement with the recent finding that individual retained introns are associated with an accumulation of the elongating form of RNA polymerase II [8].

We created an online database of IR calculated in these samples, which is available at IRBase (http://mimirna.centenary.org.au/irfinder/database).

### IR is associated with reduced protein output

**Fig. 2:**
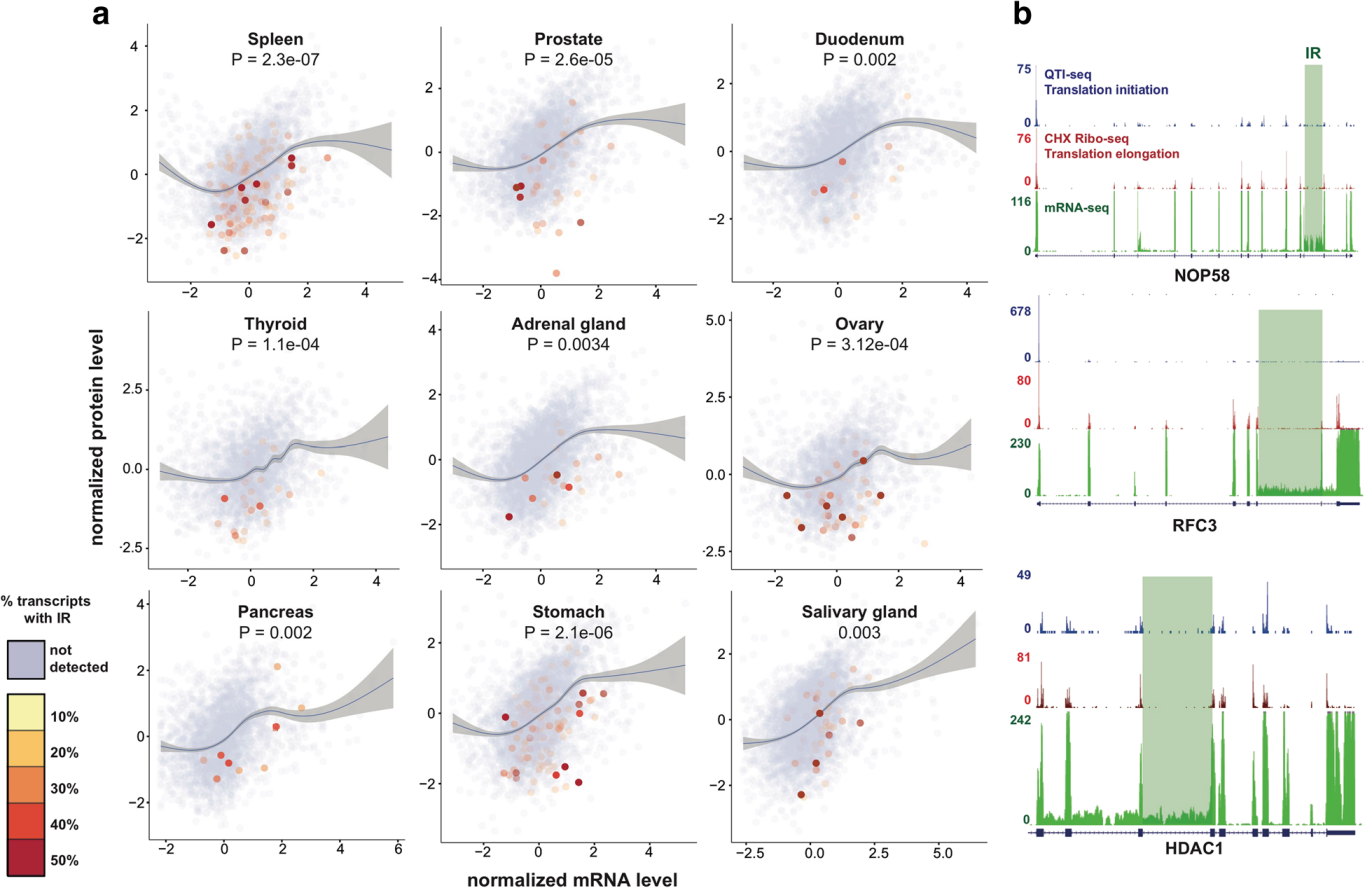
IR events alter protein output. **a** Genes with IR have significantly lower protein output (*p* values are from multivariate anova comparing mRNA and protein levels between IR and non-IR). Protein and mRNA levels normalized using a standard transformation for nine tissue types. A lowess regression curve shows the correlation between protein and mRNA levels. Genes with IR events are colored according to the maximum IR ratio of retained genes. This ratio reflects the percentage of transcripts that retain introns for a specific gene. **b** IGV screenshots of CHX Ribo-seq, QTI-seq, and mRNA-seq. Gene structure is displayed below each graph with *horizontal lines* indicating introns and *blocks* indicating exons. The sequencing coverage (number of reads) is displayed to the left. IR regions are highlighted in *green*

We previously discovered that IR coupled with NMD could dramatically reduce protein output in granulopoiesis [6]. To determine the impact of IR on protein output in multiple tissue types, we compared protein levels measured by antibody-based profiling with matched mRNA-Seq samples for nine tissue types (Fig. 2a). These data were taken from the human protein atlas [11] and analyzed for IR using IRFinder. Protein and mRNA levels were normalized using a standard score transformation (Additional file 5). Genes with IR were significantly below the regression curve, meaning that they had lower protein output than non-intron-retaining genes. Importantly, genes with IR >30% were nearly always below the regression curve, indicating that high levels of IR are nearly always associated with a lower protein output. Although this dataset is restricted by the number of proteins measured, it demonstrated that IR could reduce protein output of hundreds of genes in multiple tissue types, thereby generalizing our previous findings.

To determine whether retained introns were translated, we analyzed ribosome profiling data based on deep sequencing of ribosome-protected mRNA fragments using cycloheximide to stabilize all translating ribosomes (CHX Ribo-seq). These data were coupled with mRNA sequencing and quantitative translation initiation sequencing (QTI-seq), which uses lactimidomycin to preserve initiating ribosomes and puromycin to deplete elongating ribosomes [12]. These data allowed us to simultaneously assess translation from all translating ribosomes (CHX Ribo-seq), or specifically from initiating ribosomes (QTI-seq), and IR in HEK293 cells (Fig. 2b; Additional file 5). We found 429 IR events (IR ratio >10%) in this dataset. None of these displayed a signal from the CHX Ribo-seq or QTI-seq above background levels. This suggests that even though intron-retaining transcripts are polyadenylated, the translation machinery does generally not translate the IR elements either because the ribosome dissociates before it reaches the IR sequence or because intron-retaining transcripts are made unavailable.

### IR is associated with cell differentiation and the cell cycle

**Fig. 3:**
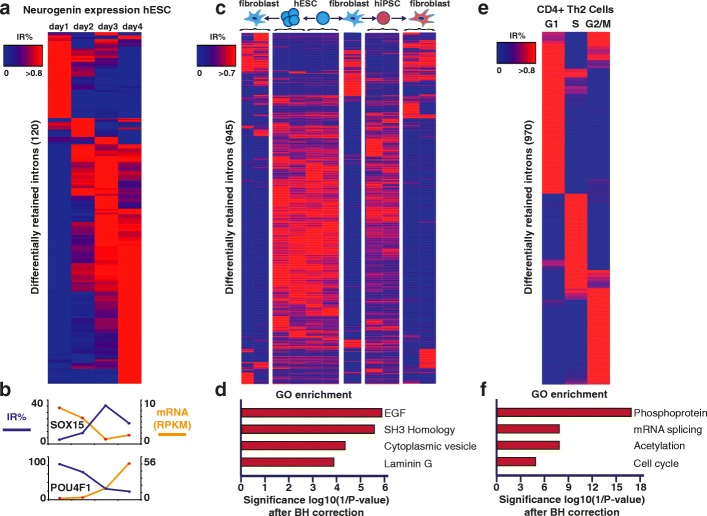
IR marks stages of cell differentiation and the cell cycle. **a** IR measured during 4 days of overexpression of Neurogenin-1 and -2 to differentiate human embryonic stem cells (*hESCs*) into bipolar neurons [16]. **b** IR and mRNA expression of two crucial genes, SOX15 and POU4F1. **c** Heatmap of IR measured during differentiation of fibroblasts from genetically matched hESC and human induced pluripotent stem cells (*hiPSC*) lines. *Left*: hESCs were grown in extended culture then differentiated into fibroblasts. *Right*: hESCs are differentiated to fibroblasts, then dedifferentiated to hiPSCs and then differentiated again into fibroblasts. *Curly brackets* indicate replicates. **d** Gene Ontology (*GO*) functional category enrichment after Benjamini–Hochberg (*BH*) correction. *EGF* epidermal growth factor. **e** IR measured for TH2 cells sorted at different stages of the cell cycle. **f** GO functional category enrichment after BH correction

We previously discovered that IR was essential for granulocyte [6], erythrocyte, and megakaryocyte [13] differentiation. Here we wished to expand our findings to three recent studies on neuronal differentiation (Fig. 3a, b), human induced pluripotent stem cells (hiPSCs) and human embryonic stem cells (hESCs) [14] (Fig. 3c, d) and the effects of the cell cycle on the differentiation of naïve T cells [15] (Fig. 3e, f). Again, we focused on introns that were retained in more than 10% of transcripts (IR ratio >0.1) with at least a coverage of three reads across the entire intron after excluding non-measurable intronic regions.

hiPSCs can be differentiated into highly homogenous neurons within 4 days by overexpressing the transcription factors Neurogenin-1 and Neurogenin-2 [16]. This allowed the authors to define networks of expression at different stages of neural differentiation. We reanalyzed their data using IRFinder and discovered 120 alternatively retained introns between days 1 and 4 of neurogenin expression (Fig. 3a; Additional file 6: Table S4). These IR events clearly marked the different stages of neuron differentiation. Similar to other studies performed in various tissues [6, 17], we found that IR in the late stage of differentiation (day 4) was enriched in splicing factor genes (*p* = 2.5 × 10^−5^). We also found numerous genes involved in neurogenesis with high levels of IR amongst which were the neural transcription factors SOX15 and POU4F1. SOX15 is known to be highly expressed in undifferentiated cells and repressed upon neuron differentiation [18]. In agreement with this, we found that mRNA levels of SOX15 dropped dramatically from 9.4 to 1.3 reads per kilobase per million mappable reads (RPKM) during neurogenin-induced differentiation (Fig. 3b). Interestingly, IR levels increased dramatically from 0 to 36% at these stages, indicating that a large proportion of transcripts retained introns. These retained introns had in-frame stop codons and were thus susceptible to degradation via the NMD pathway. Another gene, POU4F1, is activated following the neurogenic phase to regulate gene expression during neuron sensory differentiation [19]. In agreement, we found that when POU4F1 expression is first induced the intron is mostly retained, but as gene expression increases IR decreases. Here also, the retained intron had an in-frame stop codon and IR coupled with NMD could account for the dramatic change in expression of POU4F1.

We then analyzed another set of data where the authors used genetically matched hESCs and hiPSCs derived from fibroblasts using the Sendai virus reprogramming method to prove that these cells are equivalent at a gene transcription level [14]. To assess whether IR was also equivalent between these cells, we applied IRFinder to the sequencing data from this study (Fig. 3c; Additional file 6: Table S5). We found distinct patterns of IR for hESCs, hiPSCs, and fibroblasts. In agreement with this previous study, we found that fibroblasts derived from hESCs and hiPSCs had similar IR patterns. Moreover, we were able to find Gene Ontology (GO) categories significantly associated with the differential IR (945 introns; Fig. 3d). The most significant of these were epidermal growth factor (EGF), Laminin G, SH3 homology, and plasma membrane. EGF is essential for fibroblast growth and differentiation and has been described as such for over four decades [20]. SH3 homology domains interact with long distance proteins that regulate the cytoskeleton [21]. Interestingly, we found 47 genes with differential IR between iPSC and hESC fibroblasts that had not been detected in the initial study (Additional file 6: Table S6).

Finally, we investigated whether IR changed dynamically during the cell cycle in CD4^+^ T cells. IR has recently been shown to be an integral regulator of T-cell activation [22] and a recent study quantified the effects of the cell cycle on a population of naive CD4^+^ T-helper cells that were induced to differentiate toward a T_H_2 subtype [15]. They used an initial set of 892 cell cycle genes to determine cell cycle heterogeneity. We reanalyzed the sequencing data using IRFinder and discovered 969 differentially retained introns with distinct patterns of retention for each stage of the cell cycle (Fig. 3e). These introns were retained from genes enriched for phosphoproteins and the cell cycle (*p* = 2E-17 and *p* = 8E-6 after Benjamini–Hochberg correction, respectively (Fig. 3f). Surprisingly, only 97 out of the 969 introns that we found to be alternatively retained belonged to genes used to determine cell cycle signatures in the initial study. This indicates that 874 retained introns could be used as new markers of cell cycle stages in sequencing data. When we measured exonic expression of genes with retained introns, the differential expression patterns were lost (Additional file 6: Figure S4), indicating that differential IR did not mirror global mRNA expression and thus could be used as a complementary analysis to discover cell cycle markers. Interestingly, genes from the mRNA splicing GO category were also found to be enriched, leading to the hypothesis of differential regulation of splicing factors and sequence-specific regulation of IR events.

### Frequently retained introns are enriched for a subset of RNA binding sites

**Fig. 4:**
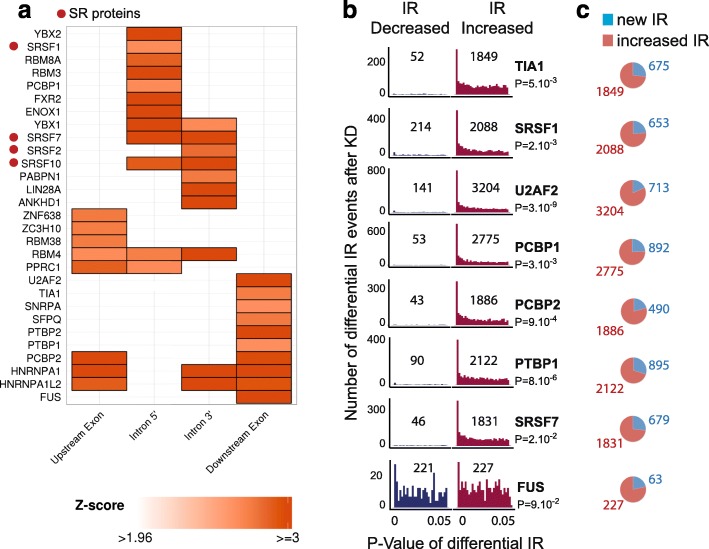
RNA binding proteins (RNAbps) modulate IR. **a** RNAbp motifs enriched (Z score >1.96) in frequently retained introns (5′ and 3′ intron boundaries) and flanking exons. No depleted motifs were found. **b** Number of increased and decreased IR events at different *p* values of significance following knockdown (*KD*). Total numbers of increased and decreased IR events are indicated on the plot. Motif enrichment *p* values in increased IR events for each RNAbp are displayed below their protein names. **c** The number of increased IR events compared to the number of retained introns following knockdown for which there was no evidence of retention before knockdown

To discover DNA motifs that could act as IR enhancers or inhibitors, we compared a set of the 1000 most frequently retained introns with 1000 rarely retained introns (Additional file 7). The most frequently retained introns were retained in over 27% of the 2573 samples analyzed, whereas rarely retained introns were retained in less than 1% of the samples analyzed. We searched for depleted or enriched k-mers between frequently retained versus rarely retained introns (Additional file 8). The regions searched comprised both flanking exons and 30 nucleotides of the 5′ and 3′ intronic boundaries. We found two clearly separated sets of enriched motifs in introns and in the flanking exons; SR protein binding sites were enriched in retained introns (Fig. 4a) and U-rich motifs in their downstream exons. We found no significantly depleted motifs in the frequently retained set. To confirm that the RNA binding motifs that were enriched contributed to increased IR, we downloaded sequencing data from the ENCODE project (https://www.encodeproject.org/) consisting of a shRNA-mediated knockdown (KD) of RNA binding proteins in HepG2 cells. We used IRFinder to calculate IR before and after KD of proteins for which the motifs were enriched in frequently retained introns (Fig. 4b). We found that IR levels increased dramatically following KD for seven out of eight RNA binding proteins. Accordingly, their binding motifs were enriched in introns for which IR increased after KD (*p* values in Fig. 4b). Interestingly, when we also searched for introns that were retained after KD but correctly spliced before, we found that there were less novel IR events than there were gains of IR levels (Fig. 4c). This indicates that a subset of RNA binding proteins attach to motifs in some introns and their flanking exons to modulate their level of regulation rather than causing novel IR events. By modulating the level of IR, they can affect the protein output through NMD. These proteins have all been studied for their role in mRNA splicing and disease [23–25] but have never been studied for their specific role in modulating IR levels. The majority of the RNA binding proteins with motifs associated with IR events are splicing regulatory factors, indicating that, to a large extent, IR events are controlled by common splicing regulatory mechanisms. Interestingly, SR proteins such as SRSF1 and SRSF7 that modulate IR are involved in splicing but also in RNA surveillance and degradation mechanisms such as NMD [26]. This suggests that the role of these and possibly other RNA-binding proteins in functions other than splicing may be mediated through their involvement in the production of IR events. Additionally, this extends to the genome scale previous models in which SR proteins auto-regulate their pre-mRNA to generate NMD targets [27].

## Conclusions

In this study we developed bioinformatics tools to study the impact of intron retention on gene regulation. Because of their length and low complexity, intron expression is difficult to measure and is affected by poorly annotated experiments. Our validated approach allowed us to measure IR in over 2573 samples to gain unique insight into the widespread nature of IR and how it affects gene regulation. We found that IR was prevalent in all samples we analyzed and that it affected over 80% of all protein coding genes making, IR a major regulator of gene expression. IR events cluster together within the same transcript and genes with IR events are closer than expected, indicating a global mechanism that regulates multiple IR events. This is in agreement with previous studies linking IR to transcription speed [8].

Given the impact of IR coupled with NMD on protein output [6], we compared protein and mRNA levels in nine tissue types and found that IR genes were significantly associated with lower protein levels. We also found no convincing evidence of translation from retained introns that escaped NMD and that were thus measurable in our samples. These transcripts may have escaped NMD because of the inefficient recruitment of UPF proteins (1, 2, or 3) to the terminating ribosome or inefficient degradation after the release of the ribosome or because they were not efficiently exported from the nucleus [17, 28, 29]. The commonly accepted role of NMD as a surveillance mechanism is supported by evidence that it prevents deleterious proteins from being created from mis-spliced transcripts in disease [30]. However, in this study and our previous study in granulopoiesis we found that none of the IR events detected created any protein products. In our model, NMD coupled with IR is a regulator of gene expression. This becomes even more apparent given that the SR proteins SRSF1 and SRSF7, which we discovered here to regulate IR levels, are also involved in NMD [26]. Not only do they enhance NMD activity but they are also associated with the exon junction complex core factors, essential for recognition by the NMD pathway [31]. In this context, the NMD machinery and SR proteins would become essential elements for a widespread mechanism of gene regulation via IR by modulating IR levels but also the efficiency of NMD that degrades IR transcripts.

We found multiple examples where IR of functionally related genes was specifically timed during differentiation and the cell cycle. Specific subsets of genes were subject to IR at different time points. Analyses of deep sequencing data in time series give a snapshot of the transcriptome. The analysis of IR may, however, be more informative than just the observation of individual transcript levels at a given time. In the data we analyzed and in our previous studies [6], we found that IR transcripts were often actively transcribed but with lower than expected protein output due to NMD. Thus, in many cases IR events often highlight genes that were required in a previous stage of differentiation or the cell cycle but are being actively downregulated. Accordingly, they give insight into the previous and future states of transcripts’ fates.

## Methods

### Novel IRFinder algorithm

The IRFinder algorithm is an improved and extended version of our first algorithm [6] and includes now tools for preparing the genome, cleaning data, and testing the suitability of a given sample for IR analysis. Many of the techniques used here can be used for analyzing other forms of splicing; however, our automated pipeline has been tuned for IR detection. Many of the cleaning and auto-detection tools described below may seem redundant given the extensive annotation of publicly available sequencing data. As we found, however, numerous samples that we collected weren’t cleaned extensively and in some cases may have been misannotated.

### Prepare genome

A STAR [32] genome index is built with a user supplied genome fasta file and annotation gtf file. An automated process allows ready use of other Ensembl genomes. All potential introns are extracted from the gtf file, being the region between two exons in any transcript. Regions covered by a gtf feature within each intron are then excluded as they are likely to confound accurate measurement of the true intron level. Excluded features are all annotations in the gtf file except those marked “retained_intron”. For directional sequencing, only features on the same strand as the intron are excluded. For non-directional sequencing, exclusions are omnidirectional.

Regions of poor unique mappability are determined by mapping synthetic reads to the genome. Synthetic reads are 70 bp single end, stepped at 10 bp across the entire reference genome. Every second read is reverse complemented. A single base error is generated in the center of each read. This error is generated in a deterministic manner, allowing reproducible results when the same input files and the same software versions are used. Reads uniquely mapping to the correct location are tallied. Any 70-bp stretch without at least five unique reads is considered poorly mappable. Poorly mappable regions are excluded from the measurable intron area regardless of strand/direction.

### Data preparation and quality controls

#### Adaptor auto-detection

Illumina sequencing, when performed on a standard paired-end library, generates two reads commencing at opposite ends of the insert and proceeding towards each other. If either read is longer than the insert, the read will continue into the sequencing adaptor. The adaptor is not reached until the entire insert is sequenced. As such, for pairs with adaptor contamination, each read commences with the insert as a reverse complementary intersection and completes with adaptor sequence at the 3′ end. We made use of this feature to automatically detect adaptors and trim them.

Our automatic detection algorithm takes the first 250,000 read pairs and performs a gapless alignment of the two reads against each other. Alignments considered have a reverse-complementary part commencing at the 5′ end with an overhanging 3′ end. From pairs with a best alignment of at least 90%, the non-overlapping components are stored as potential adaptors. This list of potential adaptors is analyzed independently for both forward and reverse reads.

Automatic detection of adaptors is implemented in a standalone PERL program and is available in the main IRFinder package. It can be readily used without dependencies both as part of this package or standalone.

### Adaptor trimming

Trimming of the adaptor, once identified, is conducted as a streaming process along with IRFinder’s mapping, count, and sort functions. The streaming process ensures unnecessary temporary files are not produced, saving both disk space and the disk I/O performance impact. Further, the streaming design substantially reduces real time to result.

Trimming uses the known adaptor sequence expected on each pair along with the complementary overlapping portion of the reads. Use of both the known adaptor sequences and the overlapping complementary section allows precise trimming, even when only one base of an adaptor is present. Trimming algorithms not using the reverse complementary segment have insufficient information for accuracy and thus must either over- or under-trim fragments containing only a short length of adaptor.

Where sequencing data are single-end only, STAR’s built-in trimming function is utilized.

Adaptor trimming is implemented as a C++ program. It can be used as an integrated part of this pipeline or standalone.

### Auto-detection of directionality

Antisense RNA can confound the calculation of IR levels, so we developed a method to automatically detect if a library was prepared using a directional protocol such as ScriptSeq or using dUTPs. These protocols enable bioinformatics analyses to correctly attribute reads to a transcript or an overlapping antisense transcript. To detect directionality, IRFinder measures the coverage across splice junctions. For each splice junction crossed by more than eight reads, if one direction is more than fourfold the other, it is counted as evidence of directionality; if not, it is evidence against directionality. If the directional score is at least 90%, the directional analysis is output for this sample. In practice the directional score is well over 99% for directional data.

### Quality control of the sample

IRFinder automatically detects samples that are not suitable for IR analysis. These samples either have high levels of DNA contamination or have been mislabeled as mRNA sequencing when in fact they are other types of experiments such as genome re-sequencing or ChIP-seq. Both DNA contamination and incorrectly labeled samples can be detected by calculating the ratio of the number of reads that map to intergenic regions to the number that maps to coding regions. If this ratio is higher than 10%, IRFinder emits a warning that this sample may not be suitable for IR detection.

To ensure reads that map to introns do not come from unprocessed mRNA, the sequencing libraries must be enriched for polyadenylated RNA. To detect RNA sequencing experiments for which the library was not enriched for mature, polyadenylated mRNAs, IRFinder counts the number of reads that map to a list of non-polyadenylated genes such as small nucleolar RNAs or specific histone genes (Additional file 9: Table S8). If the sample has not been poly(A) enriched, this read count will be much higher than expected as shown by a comparison of poly(A)- and non-poly(A)-enriched libraries (Additional file 9: Figure S6, Table S9). IRFinder will warn the user if this read count is higher than 0.01%. This threshold clearly distinguishes poly(A)- from non-poly(A)-enriched samples.

### Measuring intron retention

#### Per-sample computation

Reads are mapped to the reference genome by STAR using default scoring parameters but excluding multi-mapping reads from the output. The default scoring parameters suit the detection of IR as they favor neither mapping from exon to exon across splice junctions nor mapping from exons into introns.

**Fig. 5:**
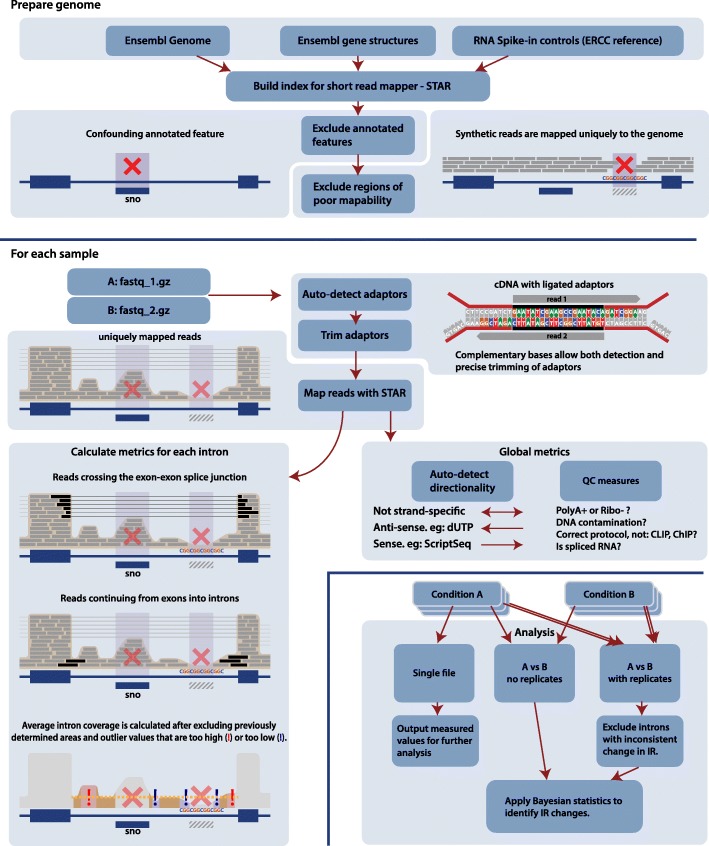
Overview of the IRFinder algorithm. *QC* quality control. *ERCC* Sequences from External RNA Controls Consortium (ERCC, http://jimb.stanford.edu/ercc/)

IR is determined by measurement of both the splicing level and intronic abundance. The key calculated metric is the IR ratio. This ratio quantifies the portion of transcription activity traversing a given intron not removed by splicing mechanisms. Abundance of normal splicing is measured by a count of read fragments spliced across the intron. Reads that start in the 5′ exon but end in another exon and reads that start in another exon but end in the 3′ exon are both counted—their sum is used as the “exonic abundance”. Intronic abundance is measured by counting the number of reads that map to an intron after having excluded features that overlap the intron and the highest and lowest 30% of values (Fig. 5). Both the exonic and intronic abundance are normalized for feature length. Normalization for library size is not required as intronic and exonic abundance are measured from the same data. The IR ratio is calculated simply as intronic abundance divided by the sum of intronic abundance and normal splicing abundance:

$$ I R- ratio=\frac{Intronic\  abundance}{\left( Intronic\  abundance+ exonic\  abundance\right)} $$

Where intron coverage is less than 1, the proportion of bases covered is used to calculate intronic abundance. The proportion of bases cannot be used as a surrogate for measuring IR ratios but is rather a means of avoiding null values when comparing an intron across multiple samples. We recommend filtering out IR candidates with coverage less than three reads across the entire measurable intron.

### Detecting changes in IR between samples

Where a pair of samples or groups is given as input to IRFinder, the final output is a listing of introns most significantly changed. Each intron can be evaluated using a Bayesian statistic adapted for digital counts [33] or DESeq2 [34] for which we have coded direct plug-ins. Significance of the change in IR ratio will be displayed in the results. Other statistics can easily be implemented on IRFinder output given the extensive information provided.

### RT-qPCR validation

To validate the results of our algorithm we used RT-qPCR to measure intron retention in two cases where IR detection can be problematic. The first validation was performed on low IR levels, the second on a long intron with regions of low mappability. Total RNA was extracted from cells using Trizol (Ambion), treated with DNaseI (Life Technologies), and converted to cDNA using Superscript III (Life Technologies). Quantification of intronic expression shown in Additional file 1 was performed with normalization against the expression of adjacent exons using RT-qPCR on the CFX96 Real-Time System (Biorad). Average deltaCT values are graphed for each cell line from three experiments. Sequences and locations of forward and reverse primers used to amplify the selected retained introns are listed in Additional file 1.

### Comparison with existing tools

To compare the efficiency of IRFinder with other existing tools that can detect IR events, we measured the average change in expression of retained introns predicted by IRFinder, MISO [35], and DEXseq [36] in a Upf2 knockout model [37].

#### Transcriptome mapping

Total RNA-seq of liver tissue from wild-type (*Upf2* ^*fl/fl*^) and Upf2 knockout (*Upf2* ^*fl/fl*^;*Mx1Cre*) mouse (GSE26561) was downloaded from the Gene Expression Omnibus (GEO). The single-end reads from both samples were mapped to the transcriptome annotation version 73 of Ensembl mouse genome GRCm38 using STAR (version and options described in IRFinder methods).

#### IRFinder

IRFinder with default parameters was applied to determine differentially retained introns between the wild-type and UPF2 knockout samples.

#### DEXSeq

DEXSeq 1.14.0 with default parameters was applied to determine differential intron usage between the wild-type and UPF2 knockout samples. Each intron with q value (false discovery rate-adjusted *p* value) less than 0.05 in the DEXSeq report was recognized as differentially retained.

#### MISO

MISO 0.5.3 with default parameters was applied to determine differentially retained introns between the wild-type and UPF2 knockout samples. Each differentially retained intron in the MISO report met the following criteria: 1) the Bayesian factor was above 19 (the likelihood that this intron is retained is 19 times higher than that of not being retained); 2) at least one read covered this intronic region; 3) at least ten reads covered the two flanking exons of this intron.

## Additional files

Additional file 1: RT-qPCR validation of IR events predicted by IRFinder. (DOCX 198 kb)
Additional file 2: Comparison of IRFinder with DEXSeq and MISO. (DOCX 86 kb)
Additional file 3: Intra- and intergeneic proximity of IR events between each other. (DOCX 60 kb)
Additional file 4: Splice site strength of IR events. (DOCX 618 kb)
Additional file 5: Comparison of IR with protein output. (DOCX 104 kb)
Additional file 6: IR during differentiation and the cell cycle. (DOCX 942 kb)
Additional file 7: List of intronic coordinates used to detect enriched motifs associate with IR. (XLS 180 kb)
Additional file 8: Method used to detect enriched motifs associate with IR. (DOCX 50 kb)
Additional file 9: Automatic detection of poly(A)-enriched samples. (DOCX 100 kb)
