# Supplementary material for: IRFinder: assessing the impact of intron retention on mammalian gene expression
Source: Genome Biol. 2017 Mar 15;18:51. doi: 10.1186/s13059-017-1184-4 (PMC5353968; doi:10.1186/s13059-017-1184-4)
Supplement: Additional file 1: — RT-qPCR validation of IR events predicted by IRFinder. (DOCX 198 kb) [file 13059_2017_1184_MOESM1_ESM.docx]

# RT-qPCR validation

The coverage across retained introns is often much lower than across exons and thus small changes in mappability, the presence of poorly trimmed reads or contaminating features can confound commonly used measures of transcript abundance. As described in the methods, IRFinder attempts to correct many of these factors to report reliable IR ratios. Our algorithm has been successfully used to dissect the function of IR in a model of granulocytic differentia-tion and in a model of induced pluripotent stem cell differentiation (Wong et al., 2013, Hussein et al., 2014).

Here we specifically validate our approach using RT-qPCR on 2 introns where IR detection can often be problematic: an intron with low coverage and an intron with regions of low mappability (Figure S1). We used probes spanning the 5′ and 3′ exon-intron boundaries as well as a probe in the middle of the intron for both introns (Table S1). We first compared IRFinder results with RT-qPCR for a retained intron expressed at a low level in the ZMIZ2 gene in human testes, K562 cells and HT29 cells (Figure S1A-C). We found that the RT-qPCR results agreed with IRFinder in all 3 tissues where a low level of IR was predicted in all 3 tissues. We then tested the last intron of PRKCA that has multiple regions of low mappability (Figure S1D-E). These regions of low mappability are visible in Figure S1D (highlighted in red) where sudden drops in coverage occur in the last intron. This intron was predicted to be retained in HT29 but not in testis or K562. These predictions were verified by RT-qPCR.


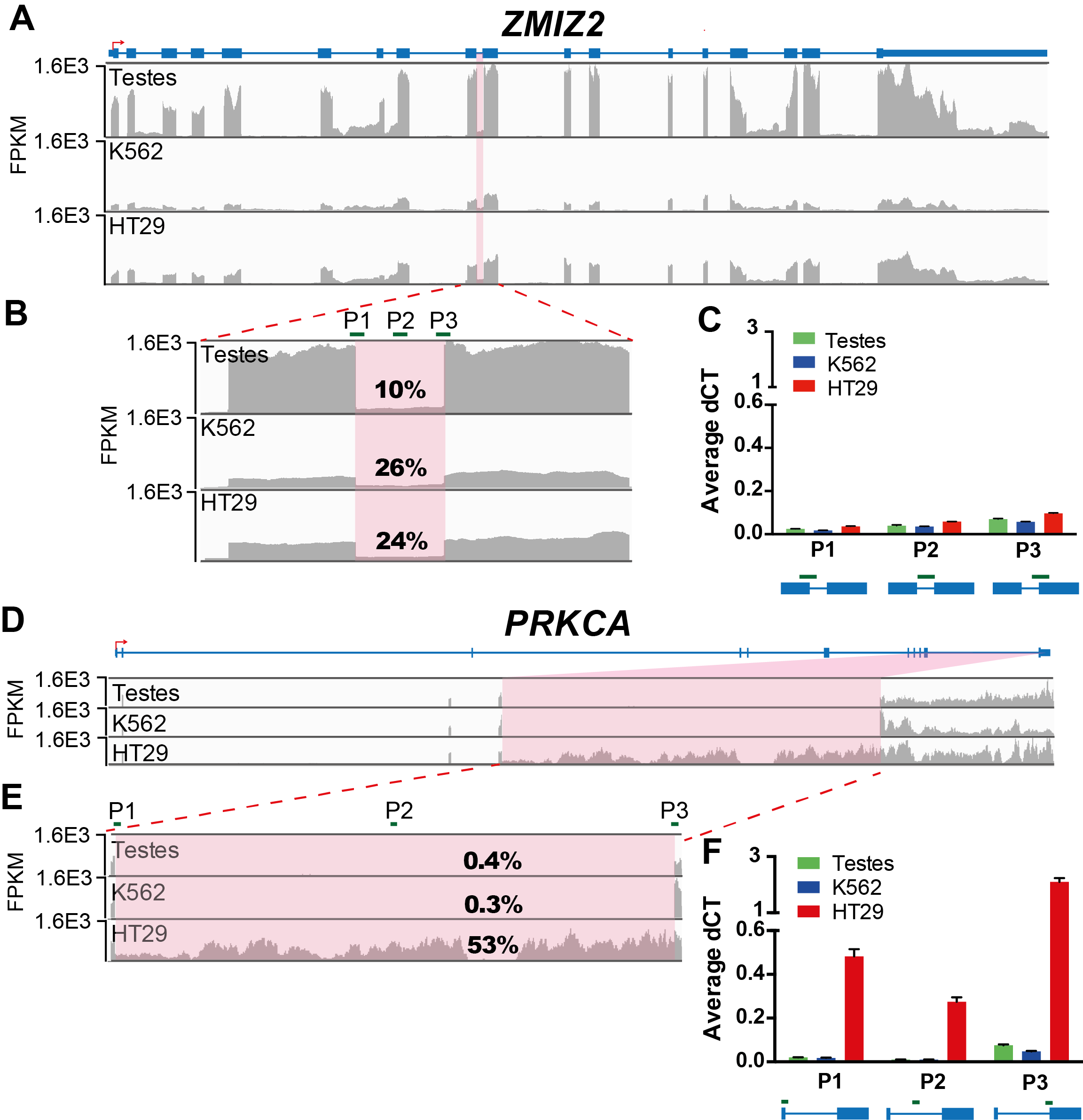


Figure S1: mRNA-seq reads (normalised per total number of mappable reads, RPM) in human testes, K562 cells and HT29 cells represented on the same scale using IGV v2.1 for *ZMIZ2* (A) and *PRKCA* (D). (B, E) Insert of region showing IR and primer positions to detect the exon-intron junction, P1; intron-retaining region, P2; and intron-exon junction, P3, expression. The IR ratio detected by IRFinder is indicated on the IGV plot. (C, F) Expression of retained introns and flanking junctions for each gene. RT-qPCR data are from three independent experiments each in triplicate (mean ± SEM).

Table S1 : Primer sequences and locations

| **Gene** | **Position** | **Primer Sequence** | **Location** |
| --- | --- | --- | --- |
| ZMIZ2 | Ex-In | TGGCTGTAAGCAACCATGTC | 7:44801133-44801250 |
|  |  | GTGTGGGAGGAGGAACAGAG |  |
|  | In-Ex | TGTTCCTCCTCCCACACTCT | 7:44801234-44801334 |
|  |  | CTCGTGGTGGTAGCACTTGA |  |
|  | In-In | GACCCTGATAATGAGGTGAGC | 7:44801179-44801298 |
|  |  | AGGCCTGCTCAGGAAGATG |  |
|  | Ex-Ex | TGGCTGTAAGCAACCATGTC | 7:44801133-44801334 |
|  |  | CTCGTGGTGGTAGCACTTGA |  |
| PRKCA | Ex-In | CTTCTTCCGGAGGATCGACT | 17:64785028-64785122 |
|  |  | AAACAGGCTGCTTTTCTGGA |  |
|  | In-Ex | CCGCATTGTCATGTTGACTG | 17:64799940-64800054 |
|  |  | GTGGTGTTAAGACGGGCTGT |  |
|  | In-In | CTGGTCTCAGGGAGTTCAGC | 17:64793857-64793950 |
|  |  | ACGATGGGTATGGGACAGAG |  |
|  | Ex-Ex | TGGAGAACAGGGAGATCCAG | 17:64785057-64800054 |
|  |  | GTGGTGTTAAGACGGGCTGT |  |
