# Supplementary material for: IRFinder: assessing the impact of intron retention on mammalian gene expression
Source: Genome Biol. 2017 Mar 15;18:51. doi: 10.1186/s13059-017-1184-4 (PMC5353968; doi:10.1186/s13059-017-1184-4)
Supplement: Additional file 2: — Comparison of IRFinder with DEXSeq and MISO. (DOCX 86 kb) [file 13059_2017_1184_MOESM2_ESM.docx]

# Comparison using simulation data.

IRFinder: IRFinder with default parameters was used.

DEXSeq: DEXSeq 1.14.0 with default parameters was used. Each intron with q value (FDR adjusted p value) less than 0.05 in the DEXSeq report was recognised as differentially retained introns.

MISO: MISO 0.5.3 with default parameters. Each differentially retained intron in the MISO report met the following criteria: 1) the Bayesian factor was above 10 (the likelihood that this intron is retained is 19-times higher than that of not being retained); 2) at least 1 read covered this intronic region; 3) at least 10 reads covered the two flanking exons of this intron.

We first generate a FASTA file to simulate ~30 million paired-end RNA-seq reads in the human genome (hg19) using the BEERS algorithm with default settings (1). These simulated reads were mapped to the Ensembl human transcriptome annotation GCRh37 Release 75 using default settings of STAR. Reads that mapped to chromosome 1 were retained for the downstream analysis. In order to show that our algorithm is robust to library size changes, we doubled the genomic coverage on chromosome 1 during the second simulation compared to retained reads of the first simulation. Upon this doubling process, we further enforced and changed intron expression patterns of 100 introns within 8 genes (Figure S2, Table S2) to simulate the expression changes of the selected intronic regions. Finally, we artificially created 40 differentially retained introns and 60 non-differentially retained introns (Supplementary Table S2). For differentially retained introns (positive control), enforced reads after doubling should cover most of the entire intronic regions and show a significant increase of expression compared to the first simulation. For non-IR events (negative control), reads could map to discrete regions within intronic regions, be aggregated around alternative 5’ splicing donor sites, be restricted to the middle regions of introns or have no enforced reads at all after doubling. We then applied IRFinder, DEXSeq and MISO on this two simulation data sets of human chromosome 1 and examined their performance based on recalling rate of these 40 positive and 60 negative controls.

IRFinder successfully identified 18 true positives while no false positives were recalled (Table S3). In contrast, although DEXSeq identified 2 less true positive compared to IRFinder, it also brought in 18 false positives. This observation is similar to its performance on the Upf2-KO dataset, indicating a low specificity of DEXSeq (Table S3). Interestingly, we also found MISO only picked up 5 true positive results with no false positive (Bayes factor cutoff of 10, Table S3). This is also similar to the observation on the real Upf2-KO dataset, where MISO found 5 times less IR events than that of IRFinder2. This might suggest that the stringent Bayes model MISO uses for identifying alternative splicing are not applicable for IR detection.


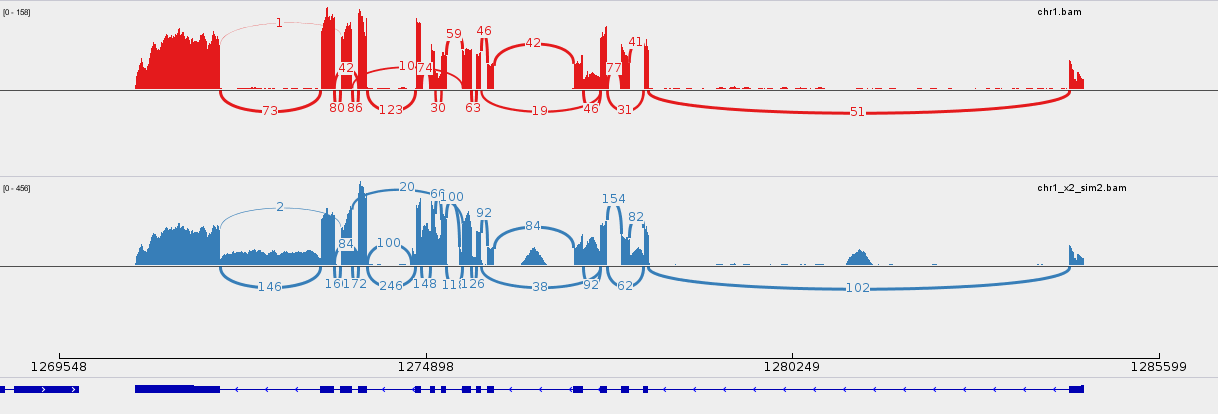


**Figure S2: Example of Simulation of differential IR within *Dvl1*.**

The first simulation by the BEERS algorithm created reads cover *Dvl1*. During the second simulation, the overall reads coverage was enforced to be doubled. For intron 1, 3, 5, 6, 8, 11 and 13 (ordered left to right), reads were further enforced to cover the whole intronic regions, upon the doubling process. These eventually generated 7 positive controls for differentially retained introns in *Dvl1*. For intron 2 and 9, small amounts of reads were enforced to cover random sites within these two intronic regions, upon the doubling process. For intron 4 and 7, reads were enforced to map to 5’ alternative splicing donor sites, upon the doubling process. For intron 10 and 14, reads were enforced to map to the middle of these two intronic regions, upon the doubling process. And for intron 12, no read was added to this intronic region after doubling. Together, intron 2, 4, 7, 9, 10, 12 and 14 made up 7 negaitve controls within *Dvl1*.

| **Table S2: Description BEERS simulation for 100 intron controls on human chromosome 1** | | | | | |
| --- | --- | --- | --- | --- | --- |
| **Chromosome** | **Start** | **End** | **Name** | **Positive/Negative Control** | **Detection** |
| 1 | 1271896 | 1273356 | dvl1_intron_1 | T | MISO,DEXSeq,IRFinder |
| 1 | 1273564 | 1273648 | dvl1_intron_2 | FS |  |
| 1 | 1273817 | 1273901 | dvl1_intron_3 | T |  |
| 1 | 1274034 | 1274741 | dvl1_intron_4 | FR | DEXSeq |
| 1 | 1274820 | 1274961 | dvl1_intron_5 | T | DEXSeq,IRFinder |
| 1 | 1275030 | 1275115 | dvl1_intron_6 | T | DEXSeq,IRFinder |
| 1 | 1275193 | 1275417 | dvl1_intron_7 | FR | DEXSeq |
| 1 | 1275558 | 1275626 | dvl1_intron_8 | T | IRFinder |
| 1 | 1275697 | 1275789 | dvl1_intron_9 | FS |  |
| 1 | 1275884 | 1277046 | dvl1_intron_10 | FM |  |
| 1 | 1277186 | 1277432 | dvl1_intron_11 | T |  |
| 1 | 1277537 | 1277742 | dvl1_intron_12 | F | DEXSeq |
| 1 | 1277865 | 1278068 | dvl1_intron_13 | T | DEXSeq |
| 1 | 1278139 | 1284275 | dvl1_intron_14 | FM | DEXSeq |
| 1 | 2440500 | 2441300 | pank4_intron_1 | FS | DEXSeq |
| 1 | 2441370 | 2441495 | pank4_intron_2 | T | IRFinder |
| 1 | 2441597 | 2442116 | pank4_intron_3 | FM |  |
| 1 | 2442222 | 2442776 | pank4_intron_4 | FS |  |
| 1 | 2442827 | 2443066 | pank4_intron_5 | FS |  |
| 1 | 2443123 | 2444326 | pank4_intron_6 | FR | DEXSeq |
| 1 | 2444479 | 2445440 | pank4_intron_7 | FM |  |
| 1 | 2445529 | 2445792 | pank4_intron_8 | T | DEXSeq,IRFinder |
| 1 | 2445906 | 2447000 | pank4_intron_9 | T |  |
| 1 | 2447157 | 2449602 | pank4_intron_10 | T | DEXSeq |
| 1 | 2449704 | 2449954 | pank4_intron_11 | T | DEXSeq,IRFinder |
| 1 | 2450037 | 2450581 | pank4_intron_12 | T | DEXSeq,IRFinder |
| 1 | 2450764 | 2451239 | pank4_intron_13 | FR |  |
| 1 | 2451394 | 2451760 | pank4_intron_14 | FR | DEXSeq |
| 1 | 2451854 | 2452161 | pank4_intron_15 | T | IRFinder |
| 1 | 2452346 | 2452539 | pank4_intron_16 | T |  |
| 1 | 2452755 | 2453156 | pank4_intron_17 | T |  |
| 1 | 2453240 | 2457902 | pank4_intron_18 | FM |  |
| 1 | 16451816 | 16455928 | epha2_intron_1 | T | DEXSeq |
| 1 | 16456085 | 16456720 | epha2_intron_2 | FR | DEXSeq |
| 1 | 16456915 | 16458215 | epha2_intron_3 | FR |  |
| 1 | 16458366 | 16458558 | epha2_intron_4 | T | MISO,DEXSeq,IRFinder |
| 1 | 16458769 | 16458872 | epha2_intron_5 | F |  |
| 1 | 16458935 | 16459674 | epha2_intron_6 | T |  |
| 1 | 16459864 | 16459975 | epha2_intron_7 | T | DEXSeq |
| 1 | 16460102 | 16460354 | epha2_intron_8 | T |  |
| 1 | 16460411 | 16460962 | epha2_intron_9 | T | IRFinder |
| 1 | 16461063 | 16461530 | epha2_intron_10 | T |  |
| 1 | 16461685 | 16462149 | epha2_intron_11 | FS |  |
| 1 | 16462266 | 16464347 | epha2_intron_12 | FR |  |
| 1 | 16464681 | 16464769 | epha2_intron_13 | T | MISO,IRFinder |
| 1 | 16464926 | 16474872 | epha2_intron_14 | FM | DEXSeq |
| 1 | 16475543 | 16477390 | epha2_intron_15 | FM |  |
| 1 | 16477459 | 16482342 | epha2_intron_16 | FM | DEXSeq |
| 1 | 21071585 | 21072035 | hp1bp3_intron_1 | T | MISO,DEXSeq,IRFinder |
| 1 | 21072150 | 21074036 | hp1bp3_intron_2 | FR | DEXSeq |
| 1 | 21074149 | 21076215 | hp1bp3_intron_3 | FR | DEXSeq |
| 1 | 21076376 | 21083658 | hp1bp3_intron_4 | FR |  |
| 1 | 21083750 | 21091869 | hp1bp3_intron_5 | FM | DEXSeq |
| 1 | 21092025 | 21094076 | hp1bp3_intron_6 | FM | DEXSeq |
| 1 | 21094158 | 21097422 | hp1bp3_intron_7 | FM | DEXSeq |
| 1 | 21097567 | 21099943 | hp1bp3_intron_8 | T |  |
| 1 | 21100104 | 21103089 | hp1bp3_intron_9 | T |  |
| 1 | 21103244 | 21106304 | hp1bp3_intron_10 | FS |  |
| 1 | 21106405 | 21106837 | hp1bp3_intron_11 | FS |  |
| 1 | 21107034 | 21113141 | hp1bp3_intron_12 | T |  |
| 1 | 23707968 | 23710838 | tcea3_intron_1 | T |  |
| 1 | 23710911 | 23713765 | tcea3_intron_2 | FM |  |
| 1 | 23713913 | 23720371 | tcea3_intron_3 | FR |  |
| 1 | 23720525 | 23724035 | tcea3_intron_4 | FR |  |
| 1 | 23724095 | 23724284 | tcea3_intron_5 | T | DEXSeq,IRFinder |
| 1 | 23724449 | 23735156 | tcea3_intron_6 | FM |  |
| 1 | 23735220 | 23743741 | tcea3_intron_7 | FM |  |
| 1 | 23743884 | 23744396 | tcea3_intron_8 | FS |  |
| 1 | 23744503 | 23745569 | tcea3_intron_9 | FS |  |
| 1 | 23745633 | 23751057 | tcea3_intron_10 | T |  |
| 1 | 35331903 | 35332648 | dlgap3_intron_1 | T | MISO,IRFinder |
| 1 | 35332793 | 35333136 | dlgap3_intron_2 | T | IRFinder |
| 1 | 35333229 | 35334205 | dlgap3_intron_3 | T |  |
| 1 | 35334691 | 35350578 | dlgap3_intron_4 | FM |  |
| 1 | 35350665 | 35351078 | dlgap3_intron_5 | FS |  |
| 1 | 35351393 | 35351672 | dlgap3_intron_6 | FS |  |
| 1 | 35351887 | 35365269 | dlgap3_intron_7 | FM |  |
| 1 | 35365343 | 35365668 | dlgap3_intron_8 | FS |  |
| 1 | 35365875 | 35369877 | dlgap3_intron_9 | FR |  |
| 1 | 35371036 | 35372955 | dlgap3_intron_10 | FR |  |
| 1 | 35373039 | 35395051 | dlgap3_intron_11 | T |  |
| 1 | 36883857 | 36884591 | oscp1_intron_1 | FS |  |
| 1 | 36884656 | 36886076 | oscp1_intron_2 | T |  |
| 1 | 36886217 | 36887750 | oscp1_intron_3 | T | DEXSeq |
| 1 | 36887821 | 36888368 | oscp1_intron_4 | T | IRFinder |
| 1 | 36888498 | 36888963 | oscp1_intron_5 | T | DEXSeq,IRFinder |
| 1 | 36889068 | 36897402 | oscp1_intron_6 | FM | DEXSeq |
| 1 | 36897484 | 36898022 | oscp1_intron_7 | FS |  |
| 1 | 36898191 | 36904356 | oscp1_intron_8 | FR | DEXSeq |
| 1 | 36904512 | 36915858 | oscp1_intron_9 | FR | DEXSeq |
| 1 | 26288575 | 26299048 | pafah2_intron_1 | F |  |
| 1 | 26299204 | 26300970 | pafah2_intron_2 | F |  |
| 1 | 26301142 | 26303172 | pafah2_intron_3 | F |  |
| 1 | 26303265 | 26308854 | pafah2_intron_4 | F |  |
| 1 | 26308969 | 26310436 | pafah2_intron_5 | F |  |
| 1 | 26310579 | 26310950 | pafah2_intron_6 | F |  |
| 1 | 26311020 | 26314721 | pafah2_intron_7 | F |  |
| 1 | 26314819 | 26315938 | pafah2_intron_8 | F |  |
| 1 | 26316093 | 26317217 | pafah2_intron_9 | T | DEXSeq |
| 1 | 26317355 | 26324515 | pafah2_intron_10 | F |  |
|  |  |  |  |  |  |
| **T: Positive control; differentially retained introns** | | | | | |
| **F: Negative control; no enforced reads after doubling** | | | | | |
| **FR: Negative control; enforced reads aggregating around 5' alternative splicing site donors** | | | | | |
| **FS: Negative control; enforced reads scattering within introns** | | | | | |
| **FM: Negative control; enforced reads aggregating at the middle of introns** | | | | | |

**Table S3: comparison of 3 algorithms on simulated data**

| **Name** | **Runtime** | **False Positive** | **True Negative** | **False Negative** | **Accuracy** | **Precision** | **Sensitivity** | **Specificity** | **FDR** |
| --- | --- | --- | --- | --- | --- | --- | --- | --- | --- |
| IRFinder | 2 | 0 | 60 | 22 | 0.78 | 1 | 0.45 | 1 | 0 |
| DEXSeq | 40 | 18 | 42 | 24 | 0.58 | 0.47 | 0.4 | 0.7 | 0.3 |
| MISO | 17 | 0 | 60 | 35 | 0.65 | 1 | 0.125 | 1 | 0 |

**Comparison using Upf2 KO data.**

Very few retained introns have been validated experimentally so it is difficult to directly compare the performance of different algo-rithms that detect IR events. However, we previously reported many retained introns that contain a premature termination codon and are candidates for degradation via the NMD pathway. Thus, by knocking out Upf2, a core component of the NMD pathway, the expression of introns from IR increases globally as these are no longer subject to NMD surveillance. We utilised IRFinder, MISO and DEXSeq to detect retained introns in mRNA-seq data from Upf2 knockout mouse liver (Upf2fl/fl;Mx1Cre) and control (Upf2fl/fl) (Weischenfeldt et al., 2012). We then compared the average change in intronic expression of predicted IR events after Upf2 knockout for all three algorithms. We found that IR events predicted by IRFinder had the highest change in expression following Upf2 knockout and predicted nearly 5 times more events than MISO which showed the second highest change in intronic expression (Table S3). Moreover, once the sequences were mapped IRFinder took less than 2 minutes to complete its predictions whereas DEX-seq took 40 minutes and MISO 17 minutes (Table S3).

1. Grant, G.R., Farkas, M.H., Pizarro, A.D., Lahens, N.F., Schug, J., Brunk, B.P., Stoeckert, C.J., Hogenesch, J.B. and Pierce, E.A. (2011) Comparative analysis of RNA-Seq alignment algorithms and the RNA-Seq unified mapper (RUM). *Bioinformatics*, **27**, 2518-2528.
