# Supplementary material for: IRFinder: assessing the impact of intron retention on mammalian gene expression
Source: Genome Biol. 2017 Mar 15;18:51. doi: 10.1186/s13059-017-1184-4 (PMC5353968; doi:10.1186/s13059-017-1184-4)
Supplement: Additional file 3: — Intra- and intergeneic proximity of IR events between each other. (DOCX 60 kb) [file 13059_2017_1184_MOESM3_ESM.docx]

# Distance between adjacent IR events

We randomly selected 30 samples in our dataset and performed the following calculations:

**Distance between genes with IR events.** A list of IR genes was created by considering genes with at least one IR event that had an IR ratio > 0.1. We then calculated the distance between each of these genes and its closest neighbour. The random control was done by selecting the same number of genes as there were IR genes amongst a list of genes that were expressed (>3 FPKM) in the sample.

**Distance between IR events within the same gene.** We considered IR events that had an IR ratio > 0.1. We then calculated the within the same gene (according to their ENSEMBL identifier) the number of IR events that were seperated by one exon. The random control was done by randomly selecting the same number of introns for each gene amongst introns annotated in our GTF file.
