# Supplementary material for: IRFinder: assessing the impact of intron retention on mammalian gene expression
Source: Genome Biol. 2017 Mar 15;18:51. doi: 10.1186/s13059-017-1184-4 (PMC5353968; doi:10.1186/s13059-017-1184-4)
Supplement: Additional file 4: — Splice site strength of IR events. (DOCX 618 kb) [file 13059_2017_1184_MOESM4_ESM.docx]

# IR enhancer motifs and splicing site strength

We analysed splice site strength using MaxEntScan(1) . Following the authors recommendations we calculated a MaxEntScore based on maximum entropy of 9bp at the 5’ (donor) sites and 23 bases at the 3’ (acceptor) sites. The 3’ acceptor site scores were weakly significantly different (P=0.002; Kolmogorov-Smirnov test) and the 5’ donor sites displayed a significant difference (P=5.10^-7^; Kolmogorov-Smirnov test), between retained and non-retained groups (medians of 8.5 and 9 respectively).

**Figure S3: splice site strength around frequently spliced introns (blue) and frequently retained introns (red.)**

Motif analysis was performed by detecting enriched oligonucleotides relative to control datasets. As controls, 1000 introns that were least frequently retained were used. Subsequently, for each control, the enrichment of oligonucleotide sequences of length k, i.e. k-mers, was measured in two different ways: 1) Comparing the overall frequencies of k-mers in retained introns versus the frequencies from the control set, and 2) comparing the proportion of retained introns with a given k-mer versus the proportion of control introns with that k-mer. In both cases a z-score was calculated by comparing the observed value with the distribution of the control introns for each k-mer. Motif analysis of the 5' and 3' splice-sites are performed separately, to assess splice-site motifs independently of other possible splicing regulatory motifs.

1. Yeo, G. and Burge, C.B. (2004) Maximum entropy modeling of short sequence motifs with applications to RNA splicing signals. *J Comput Biol*, **11**, 377-394.
