# Supplementary material for: IRFinder: assessing the impact of intron retention on mammalian gene expression
Source: Genome Biol. 2017 Mar 15;18:51. doi: 10.1186/s13059-017-1184-4 (PMC5353968; doi:10.1186/s13059-017-1184-4)
Supplement: Additional file 5: — Comparison of IR with protein output. (DOCX 104 kb) [file 13059_2017_1184_MOESM5_ESM.docx]

# IR and protein output

**IR and protein levels**

Protein and mRNA data were downloaded from <http://www.proteinatlas.org/about/download>. Using their protocol, reads were mapped to the human genome (GRCh37) using Tophat v2.0(1). FPKM (fragments per kilobase of exon model per million mapped reads) values were calculated using Cufflinks v2.1(2). FPKM values were normalized within each sample by using the following standrdized score transformation:


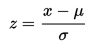


**Ribo-seq, QTI-seq and IR**

mRNA-seq data was downloaded from experiment ID SRR1630838 and analysed using IRFinder. We found 429 IR events where IR levels were > 10%. QTI-seq and Ribo-seq data were downloaded from and analysed by the GWIPS website(3). We selected IR sequences with at least 20 reads covering it in Ribo-seq. After exclusion of initiation peaks that overlapped small transcription elements such as snoRNAs or microRNAs, QTI-seq did not reveal any translation initiation sites in the IR sequences. Furthermore, Ribo-seq density in IR sequences did not exceed the background level and were undiminished in QTI-seq, excluding that these reads came from actively translating ribosomes.

1. Trapnell, C., Pachter, L. and Salzberg, S.L. (2009) TopHat: discovering splice junctions with RNA-Seq. *Bioinformatics*, **25**, 1105-1111.

2. Trapnell, C., Roberts, A., Goff, L., Pertea, G., Kim, D., Kelley, D.R., Pimentel, H., Salzberg, S.L., Rinn, J.L. and Pachter, L. (2012) Differential gene and transcript expression analysis of RNA-seq experiments with TopHat and Cufflinks. *Nature protocols*, **7**, 562-578.

3. Michel, A.M., Fox, G., A, M.K., De Bo, C., O'Connor, P.B., Heaphy, S.M., Mullan, J.P., Donohue, C.A., Higgins, D.G. and Baranov, P.V. (2014) GWIPS-viz: development of a ribo-seq genome browser. *Nucleic Acids Res*, **42**, D859-864.
