# Supplementary material for: IRFinder: assessing the impact of intron retention on mammalian gene expression
Source: Genome Biol. 2017 Mar 15;18:51. doi: 10.1186/s13059-017-1184-4 (PMC5353968; doi:10.1186/s13059-017-1184-4)
Supplement: Additional file 6: — IR during differentiation and the cell cycle. (DOCX 942 kb) [file 13059_2017_1184_MOESM6_ESM.docx]

# IR during differentiation and cell cycle

We measured alternately retained introns between different stages of differentiation using using a Bayesian statistic adapted for digital counts (1). Significant (P<0.001) changes also had to have a change in IR% of at least 10%.

**Figure S4:** Comparison between IR ratios (left) and the expression of their flanking exons (right) shows that the IR patterns define each stage of cell cycle whereas their exonic expression does not.


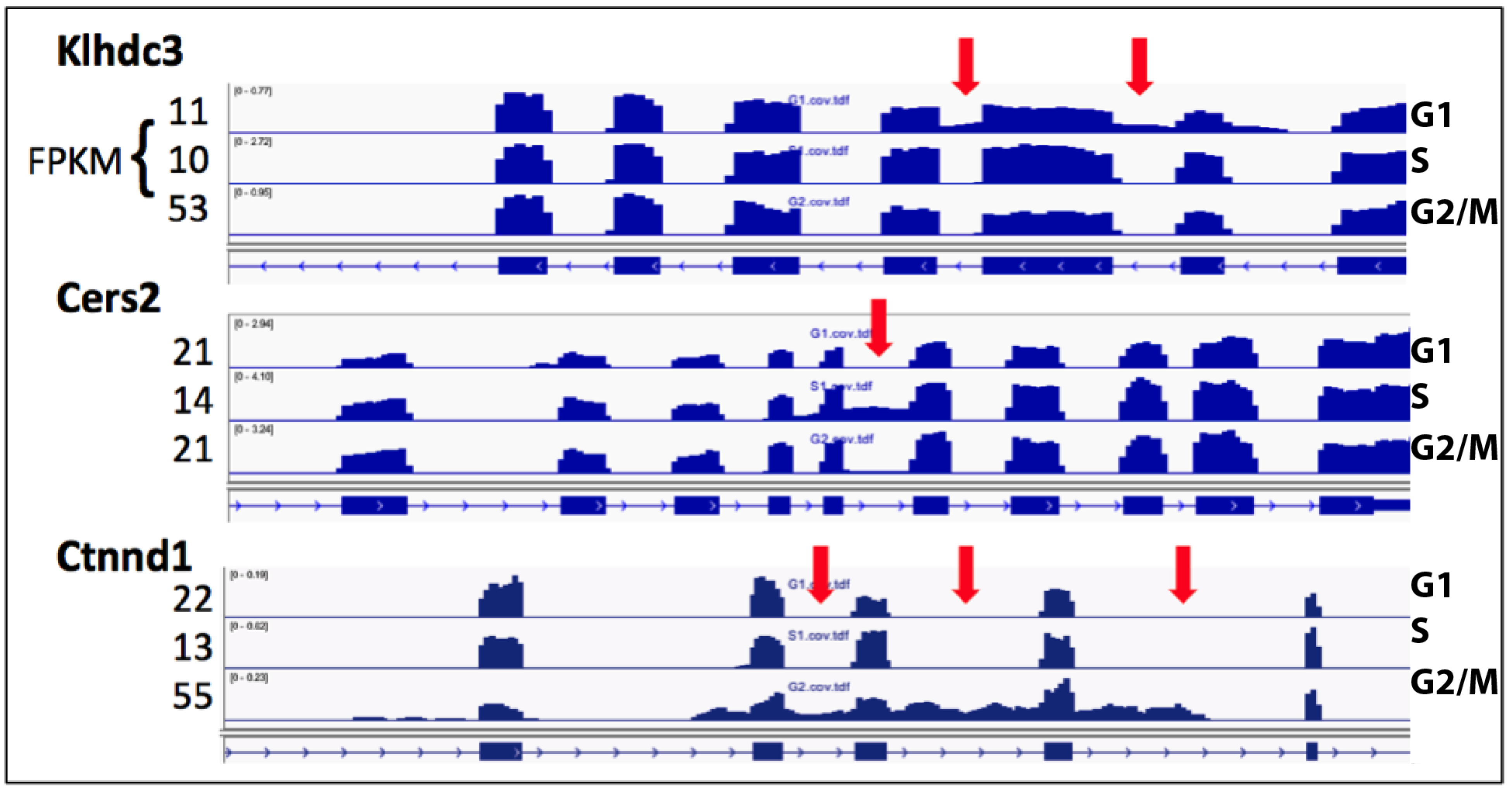


**Figure S5**: IGV screenshots showing examples of differentially retained introns in G1,S and G2 phase (from top to bottom). Arrows shows IRFinder predictions. FPKM values calculated by HTSeq have been added to the left.

**Table S4: Genes with differentially retained introns during neurogenisis**

| ABCA5 | CLEC4G | IFNLR1 | NCAPD3 | QRICH2 | TERT | ZSCAN10 |
| --- | --- | --- | --- | --- | --- | --- |
| ADAM11 | CNTNAP1 | INSRR | NDUFA3 | RAD54L | TEX10 |  |
| ADAM33 | CPNE7 | KCNAB1 | NEK8 | RECQL4 | THBS3 |  |
| ANO9 | CPSF3L | KCNIP2 | NTHL1 | RGL3 | TONSL |  |
| APC2 | DDB2 | KIAA1161 | OBSCN | RGS11 | TRIM66 |  |
| ARHGEF19 | DENND6B | KIAA1875 | OBSL1 | RHBDL1 | TSC2 |  |
| ARRDC1 | DKKL1 | KREMEN2 | OPLAH | RPS6KA4 | TTBK1 |  |
| ATL2 | DNM1 | LAMP5 | OSBP2 | SAMD11 | TUB |  |
| B3GNT8 | EFCAB10 | LARP7 | PCDH8 | SCN3B | UNC5A |  |
| BTNL9 | EME1 | LRRC45 | PER2 | SEC14L1 | VPS9D1 |  |
| C19orf33 | EXOC3L1 | MAP3K12 | PIEZO1 | SEMA4G | VWA5B2 |  |
| CARD9 | FADS2 | MAPK15 | POLR2A | SH2D6 | VWDE |  |
| CCDC61 | GABBR1 | MAST3 | POU4F1 | SLC18A2 | WDR24 |  |
| CCDC78 | GCHFR | MDGA1 | PPP3CC | SLC29A2 | WDR90 |  |
| CCDC88B | GLB1L2 | MED11 | PRDM8 | SNAP91 | XYLT2 |  |
| CDH15 | GLB1L3 | MIB2 | PSTPIP1 | SNAPC4 | YPEL4 |  |
| CDH23 | GPR123 | MLLT4-AS1 | PTCH2 | SOX15 | YRDC |  |
| CDK11B | HHATL | MMP25 | PTPRD | SUN2 | ZC3H12D |  |
| CHRNG | ICAM5 | MUC19 | PVALB | SYDE2 | ZNF57 |  |

**Table S5: Genes with differentially retained introns during iPSC, hESC, fibroblast differentiation**

| A2ML1 | BRSK1 | CRB2 | FAT4 | IL17RE | METRN | PCSK5 | RNF39 | ST6GALNAC2 | VILL |
| --- | --- | --- | --- | --- | --- | --- | --- | --- | --- |
| ABHD12B | BRSK2 | CRHR2 | FBN3 | IL23A | METTL15 | PDE6B | RRAGB | STAC3 | VIPR2 |
| ABHD14B | BTBD19 | CRIP3 | FBXO24 | INPP5E | MFSD7 | PDK1 | RYR2 | STEAP2 | VPS41 |
| AC144521.1 | BZRAP1 | CRTC1 | FBXO48 | INPP5J | MIB2 | PDZD4 | SBK2 | STK38 | VPS9D1 |
| ACBD4 | CACNA1H | CSPG4 | FBXO6 | IQSEC2 | MIR3654 | PHACTR1 | SCUBE1 | STXBP5 | VSIG10L |
| ACRBP | CACNB2 | CSRNP3 | FCHO1 | ITGA2B | MLXIPL | PHC2 | SCUBE3 | SULT1A2 | VSIG2 |
| ADAM11 | CALML4 | CTC-479C5.12 | FGFR3 | ITGA7 | MMP24 | PHF21B | SEC14L6 | SUN1 | VWDE |
| ADAM28 | CAPN11 | CTC-550B14.6 | FMNL1 | ITGB1BP2 | MMP25 | PHGDH | SEC31B | SUN2 | WDR17 |
| ADAM33 | CAPN12 | CTC1 | FOXJ3 | ITGB7 | MMRN2 | PHKA1 | SEMA4D | SYCE1L | WDR61 |
| ADAM8 | CAPN15 | CTD-2527I21.4 | FRAS1 | JAG2 | MPP4 | PHKA2 | SEMA5B | SYT6 | WDR86 |
| ADAMTS13 | CARD10 | CTD-3088G3.8 | FSD1L | JAK3 | MRVI1 | PIEZO2 | SERHL2 | SYTL4 | WDR86-AS1 |
| ADAMTS14 | CARD9 | CUBN | FSIP2 | JAKMIP2 | MST1R | PIF1 | SERPINF2 | TAPBPL | WIF1 |
| ADAMTS2 | CASKIN1 | CUL7 | FTCD | JPH1 | MTCP1 | PIGG | SFPQ | TARBP1 | WNK4 |
| ADAMTS20 | CATSPER1 | CYP27C1 | GABRQ | KATNBL1 | MTHFD2L | PIK3IP1 | SGSH | TBC1D26 | WRN |
| ADAMTSL4 | CATSPER2 | CYP2D6 | GAD1 | KAZN | MTMR3 | PILRB | SGSM1 | TBC1D8 | XDH |
| ADHFE1 | CCDC142 | CYP4V2 | GANC | KCND2 | MTMR7 | PLA2G3 | SH2D2A | TBRG1 | XKR5 |
| AGAP2 | CCDC150 | CYP4X1 | GARNL3 | KCNMB3 | MTR | PLA2G6 | SH2D3A | TBX6 | XRCC3 |
| AGER | CCDC153 | D2HGDH | GBA2 | KCP | MUC19 | PLCB4 | SH2D5 | TBXAS1 | ZBBX |
| AIM1 | CCDC154 | DAB1 | GDPD3 | KIAA1377 | MUC3A | PLEKHA6 | SH3RF2 | TEC | ZBTB49 |
| AKAP8 | CCDC169-SOHLH2 | DCDC2 | GEN1 | KIAA1407 | MX2 | PLEKHA7 | SHISA6 | TERT | ZDHHC1 |
| ALG1L | CCDC17 | DDX19B | GHDC | KIAA1755 | MYBPC2 | PLEKHH1 | SIK2 | TET2 | ZDHHC19 |
| ALOX12 | CCDC180 | DENND1C | GLB1L2 | KIF12 | MYCBPAP | PLEKHN1 | SKIDA1 | TEX9 | ZDHHC23 |
| ALPK1 | CCDC183 | DET1 | GLB1L3 | KIF25 | MYH3 | PLXNA3 | SLC13A4 | TGM1 | ZFHX3 |
| ALPPL2 | CCDC61 | DFNA5 | GLP1R | KLC3 | MYH7B | PLXNB3 | SLC15A2 | THBS3 | ZFPM1 |
| ALS2CL | CCDC65 | DGKD | GLS2 | KLF4 | MYO15A | PLXNC1 | SLC16A11 | THBS4 | ZFR2 |
| AMN | CCDC78 | DGKE | GLT8D2 | KNDC1 | MYO15B | PNKD | SLC18A2 | THNSL2 | ZIC3 |
| ANKDD1A | CCDC88B | DGKQ | GOLGA1 | KRBA1 | MYO6 | PNPLA5 | SLC1A4 | TIRAP | ZMAT1 |
| ANKLE1 | CD1D | DIP2A | GPR162 | KREMEN2 | MYO7B | POU6F1 | SLC22A3 | TLE6 | ZNF236 |
| ANKRD24 | CD47 | DIRC3 | GRB14 | L3HYPDH | NAALAD2 | PPP1R32 | SLC22A31 | TM4SF19 | ZNF275 |
| ANKRD45 | CD48 | DLEC1 | GREB1 | L3MBTL1 | NADSYN1 | PPP1R9A | SLC22A5 | TMC4 | ZNF276 |
| ANKRD61 | CDAN1 | DLL1 | GREB1L | LAMA5 | NAGS | PRICKLE4 | SLC25A28 | TMEM145 | ZNF280C |
| ANO8 | CDC42BPG | DMC1 | GRHL1 | LAMC3 | NAPEPLD | PRKCG | SLC29A4 | TMEM155 | ZNF354C |
| ANO9 | CDCA5 | DNAH11 | GRHL2 | LARP7 | NBEAL2 | PRODH | SLC2A11 | TMEM180 | ZNF418 |
| ANPEP | CDHR1 | DNAJB6 | GRIA3 | LDHD | NBPF1 | PRPF40B | SLC2A4 | TMEM194A | ZNF765 |
| AP003068.23 | CEL | DNHD1 | GRIA4 | LEFTY1 | NCR1 | PRSS45 | SLC2A8 | TMEM253 | ZNF789 |
| APC2 | CELSR3 | DNM1 | GRIK3 | LEMD2 | NDUFA6-AS1 | PSD | SLC38A3 | TMEM74B | ZNF90 |
| ARF6 | CENPC | DNMT3B | GRIN3B | LGI4 | NDUFAF6 | PSMB10 | SLC39A4 | TNK1 | ZP1 |
| ARHGAP19-SLIT1 | CENPJ | DNMT3L | GRIP1 | LIFR | NEB | PTCHD2 | SLC39A5 | TNNI1 | ZSCAN10 |
| ARHGAP40 | CEP164 | DOCK3 | GRIP2 | LINC00173 | NECAB2 | PTH1R | SLC44A5 | TNXB | ZSCAN22 |
| ARHGEF16 | CEP78 | DQX1 | GRK4 | LLGL2 | NEIL1 | PTPN5 | SLC45A3 | TONSL | ZSCAN23 |
| ARHGEF17 | CES3 | DST | GRM4 | LOXL3 | NEK8 | PTPRN | SLC4A11 | TPCN1 | ZSWIM5 |
| ARHGEF19 | CHI3L1 | DUOX1 | GRTP1 | LPXN | NFRKB | QRICH2 | SLC52A3 | TPCN2 | ZSWIM8 |
| ARHGEF26 | CHIC1 | DVL1 | GTDC1 | LRCH4 | NIPAL2 | R3HDM2 | SLC5A9 | TRAF2 | ZYG11A |
| ARHGEF40 | CHPF2 | ECEL1 | GUCA1B | LRP11 | NKAIN4 | RAB11FIP3 | SLC7A10 | TRAIP |  |
| ARL5A | CKMT2 | EDA2R | GUSBP11 | LRP2BP | NKD2 | RAB17 | SLC7A3 | TRIM46 |  |
| ARMC9 | CLASP2 | EFCAB10 | H6PD | LRRC16B | NLRP7 | RAD54L | SLC9A5 | TRIML2 |  |
| ARSD | CLCN1 | EFCAB13 | HAP1 | LRRC34 | NME8 | RALGAPA1 | SLCO1A2 | TRMT13 |  |
| ART5 | CLCN2 | EGFLAM | HEXDC | LRRC6 | NOSTRIN | RALGPS1 | SLCO4A1 | TSGA10 |  |
| ARVCF | CLCN7 | ELMO3 | HFM1 | LRRIQ1 | NOXA1 | RANBP17 | SLFN13 | TSPAN11 |  |
| ASB12 | CLCNKA | EME1 | HHIP | LRRK1 | NPL | RAPGEF4 | SLMAP | TTC12 |  |
| ASIC3 | CLCNKB | ENG | HHIPL2 | LRRTM3 | NPR1 | RAPGEF5 | SNAPC4 | TTC17 |  |
| ATAD5 | CLDN15 | ENOSF1 | HIST1H4H | LTB | NPY1R | RASAL1 | SNHG14 | TTC28 |  |
| ATG9B | CLPSL2 | ENTPD2 | HMCN2 | MAMDC4 | NUDT8 | RASGRP2 | SNTG2 | TTC40 |  |
| ATL1 | CLYBL | EPB41L4A | HPD | MAP3K10 | OARD1 | RASIP1 | SNX32 | TTN |  |
| ATP2A1 | CNKSR1 | EPN3 | HPN | MAP3K4 | OBSCN | RBM11 | SOX13 | TULP2 |  |
| ATP2C2 | CNTN2 | EPS8L1 | HPS1 | MAP4K1 | OBSL1 | RBP5 | SOX15 | UBN2 |  |
| ATP8B3 | CNTNAP1 | ESYT3 | HSBP1L1 | MAP6 | OC90 | RDH16 | SP140L | UGGT2 |  |
| ATRX | COG7 | EVC | HSD3B7 | MAPK15 | OIP5-AS1 | RELT | SPAG4 | UNC13D |  |
| AURKC | COL11A1 | EXD3 | HSPA12B | MARC1 | OPHN1 | RET | SPATA18 | UQCC1 |  |
| B3GNT9 | COL4A3 | EXOC3L1 | IDUA | MATK | OPLAH | RFTN2 | SPATA5L1 | USE1 |  |
| BACE1 | COL4A5 | EXTL1 | IFNLR1 | MCOLN2 | OSBP2 | RGL3 | SPEG | USP20 |  |
| BAI2 | COL4A6 | FAM124A | IFT140 | MCTP1 | OVGP1 | RHBDL1 | SPINK1 | USP45 |  |
| BAIAP2L2 | COLCA2 | FAM132A | IFT172 | MED12L | PADI3 | RHPN1 | SPOCD1 | USP9Y |  |
| BEST1 | COX6B2 | FAM211A | IGFL2 | MEF2B | PANK2 | RNF112 | SRCRB4D | UVSSA |  |
| BET1 | CPNE1 | FAM65C | IGSF1 | MEGF6 | PAPLN | RNF149 | SRGAP3 | VASH2 |  |
| BRICD5 | CPNE7 | FAT2 | IGSF9 | METAP1D | PCSK4 | RNF212 | SS18L1 | VENTX |  |

**Table S6: Differential IR between iPSC and hESC derived fibroblasts**

| ADAMTS20 | MMRN2 |
| --- | --- |
| ART5 | MST1R |
| ATP2C2 | MTHFD2L |
| C6orf223 | MTHFD2L |
| CAPN12 | MYCBPAP |
| CARD10 | MYH7B |
| CASKIN1 | NEB |
| CCDC88B | NOSTRIN |
| CTD-3088G3.8 | NPY1R |
| DENND1C | PDE6B |
| DNHD1 | PSD |
| DQX1 | RASIP1 |
| EPB41L4A | RBP5 |
| EXD3 | RET |
| FSIP2 | SGSH |
| GREB1L | SLC2A4 |
| GRM4 | SLC39A5 |
| HFM1 | ST6GALNAC2 |
| HSPA12B | STAC3 |
| KCP | TBC1D26 |
| LINC00173 | TMC4 |
| LRRC16B | TNXB |
| MFSD7 | TTN |
| MMP25 | ZP1 |

**Table S7: Genes with differentially retained introns during G1,S and G2/M cell cycle stages**

| 0610011F06Rik | AW146154 | Cog2 | Esyt1 | Hfm1 | Mark3 | Nup160 | Rab29 | Smek1 | Trim33 |
| --- | --- | --- | --- | --- | --- | --- | --- | --- | --- |
| 0610030E20Rik | AW549877 | Coil | Exoc1 | Hif1a | Mau2 | Nup214 | Rab34 | Smek2 | Trip12 |
| 1110007C09Rik | Axin1 | Copa | Ezh1 | Hmgxb3 | Mbip | Obsl1 | Rab3gap1 | Smg1 | Trmt2a |
| 1110038F14Rik | B4galnt4 | Coro7 | F2 | Hnrnpl | Mbtd1 | Ogfrl1 | Rab5b | Smg7 | Trpm4 |
| 1110051M20Rik | Bace1 | Cpt1c | Fam102a | Homer3 | Mdn1 | Ogt | Racgap1 | Smpd4 | Trrap |
| 1700023H06Rik | Baz1b | Creb3 | Fam126b | Hps3 | Mdp1 | Ormdl2 | Rad50 | Smurf2 | Tsga10 |
| 2310033P09Rik | Baz2a | Csnk1a1 | Fam133b | Hps5 | Med23 | Osbp | Rad54b | Snap29 | Tstd1 |
| 2610507B11Rik | Bbs4 | Csnk1d | Fam13b | Hsd3b7 | Med24 | P2rx4 | Rad54l | Snf8 | Ttc14 |
| 2810403A07Rik | Bbs7 | Csrp2bp | Fam173a | Hsf2 | Mettl17 | P4htm | Rad9b | Snrnp200 | Ttc19 |
| 4833420G17Rik | BC003965 | Ctc1 | Fam193a | Huwe1 | Mettl23 | Pan2 | Raf1 | Snx11 | Ttc25 |
| 4932438A13Rik | Bcl3 | Ctnnd1 | Fam193b | Hyal1 | Mfsd3 | Parn | Ranbp2 | Snx15 | Ttc3 |
| A230046K03Rik | Bdp1 | Ctr9 | Fam208a | Ifrd2 | Mfsd7b | Parp6 | Rangap1 | Sorl1 | Ttc39b |
| A2m | Birc6 | Ctsa | Fam21 | Ift81 | Mia3 | Pax6 | Rasa4 | Sos1 | Ttf2 |
| Abca7 | Bmi1 | Ctsf | Fam65a | Igf2bp3 | Mier3 | Paxip1 | Rassf7 | Spaca6 | Ttll3 |
| Abcd4 | Brms1 | Ctu2 | Fam76b | Ikbkap | Mlh1 | Pcf11 | Rb1cc1 | Spag5 | Ttll4 |
| Abcf3 | Brwd1 | Cul1 | Fancb | Impdh1 | Mms19 | Pcgf6 | Rbm28 | Spg11 | Tubgcp4 |
| Abhd16a | Btaf1 | Cul4a | Fanci | Incenp | Morc2a | Pcif1 | Rbm5 | Spice1 | Tubgcp5 |
| Ablim1 | Cacna1a | Cwf19l2 | Fank1 | Ino80b | Mppe1 | Pcm1 | Rbms1 | Spred3 | Uba6 |
| Acadvl | Calcoco1 | Cxxc1 | Fastk | Inpp5k | Mprip | Pcolce2 | Renbp | Sptan1 | Ubap2 |
| Acd | Cald1 | Cyp4f13 | Fbxo18 | Ints2 | Mri1 | Pcyt2 | Rfwd3 | Srcap | Ubap2l |
| Acer1 | Camsap3 | Cyth2 | Fbxo38 | Ints3 | Mrpl38 | Pdlim5 | Rfx2 | Srgap2 | Ube3a |
| Acp5 | Capn7 | D15Ertd621e | Fdxr | Ints6 | Mrpl57 | Pds5a | Rictor | Ssbp3 | Ube3c |
| Adat1 | Caprin1 | D2Wsu81e | Fibp | Ints7 | Mrps10 | Pdxdc1 | Rif1 | Ssc4d | Ubp1 |
| Adck5 | Casc5 | Dalrd3 | Fkbp15 | Ipo11 | Msln | Peo1 | Rint1 | Stag3 | Ubr3 |
| Adcy3 | Casp2 | Dars2 | Flna | Ipo9 | Mta2 | Pex1 | Ripk3 | Star | Ubxn7 |
| Adgre5 | Cbfa2t2 | Ddx17 | Fmr1 | Ireb2 | Mtf1 | Pex5 | Rmdn3 | Stat2 | Unc45a |
| Afg3l2 | Cbl | Ddx20 | Fn1 | Irf9 | Mtif2 | Pfas | Rmnd1 | Stat3 | Uri1 |
| Agfg2 | Cby1 | Ddx27 | Fnbp4 | Itfg2 | Mtmr4 | Pgam5 | Rmnd5a | Stau1 | Uso1 |
| Ago1 | Ccdc114 | Ddx31 | Fndc3a | Itga5 | Mto1 | Pgp | Rnd2 | Stk19 | Usp1 |
| Ago2 | Ccdc117 | Ddx50 | Fryl | Jmjd7 | Mtor | Phactr4 | Rnf167 | Stt3b | Usp19 |
| Ahctf1 | Ccdc134 | Ddx58 | Fsd1 | Kank3 | Mum1 | Phf12 | Rnf213 | Stx4a | Usp2 |
| Ahcyl1 | Ccdc66 | Decr2 | Fxyd4 | Kansl1 | Mus81 | Phf8 | Rnpep | Stx5a | Usp21 |
| Ajuba | Ccdc84 | Dennd1c | G6pdx | Kat5 | Mycbp2 | Phkg1 | Rock1 | Styx | Usp24 |
| Akap10 | Ccdc88a | Dennd2a | Gabbr1 | Kat6b | Myef2 | Phkg2 | Rock2 | Sult6b1 | Usp34 |
| Aldh18a1 | Cchcr1 | Dennd2c | Gamt | Kbtbd4 | Myh10 | Phtf2 | Rpap1 | Surf1 | Usp37 |
| Aldh6a1 | Ccni | Dennd5a | Gatad2b | Kctd3 | Myo18a | Phykpl | Rrbp1 | Suv39h1 | Usp53 |
| Alg1 | Ccnl1 | Dennd6a | Gclm | Kdelc1 | Myo19 | Pi4k2b | Rspry1 | Suv420h1 | Usp9x |
| Alg11 | Ccnt2 | Depdc5 | Gfm2 | Kdm2a | Myo1c | Pias3 | Rtn4 | Suz12 | Vprbp |
| Alg12 | Cd109 | Dgat1 | Ggcx | Kdm6b | Myo1e | Pick1 | Rufy1 | Syce1 | Vps33b |
| Alg13 | Cd151 | Dgcr8 | Gigyf1 | Khdrbs1 | Myo5a | Pidd1 | Saa3 | Taf1 | Vps4a |
| Alkbh8 | Cd300e | Dhrs11 | Gls | Kif14 | Myo9a | Pif1 | Sat1 | Taf1c | Vps9d1 |
| Amd1 | Cd55 | Dhx29 | Gls2 | Kif15 | Naa16 | Pip5k1a | Sat2 | Tango6 | Vwa5a |
| Ammecr1l | Cdan1 | Diap1 | Glt8d1 | Kif20b | Nagpa | Plaa | Sbno2 | Tbk1 | Wdr24 |
| Amt | Cdc14b | Dido1 | Gm166 | Kif5c | Naprt | Plcb4 | Scaf8 | Tcerg1 | Wdr34 |
| Anapc1 | Cdc25c | Dis3l | Gm17066 | Klf4 | Nbr1 | Plekha2 | Sccpdh | Tcirg1 | Wdr76 |
| Anapc2 | Cdk16 | Dlg5 | Gm29609 | Klhdc3 | Nckipsd | Plekha8 | Sct | Tcta | Whsc1 |
| Anks3 | Cdk18 | Dmtf1 | Gmpr2 | Kmt2c | Ncoa3 | Plxna3 | Sdr39u1 | Telo2 | Wrap73 |
| Ankzf1 | Cenpi | Dmxl1 | Gna11 | Kmt2e | Ncoa5 | Pm20d1 | Sec16a | Tep1 | Xpot |
| Ano9 | Cep128 | Dnah8 | Gnl3 | Kpna1 | Ndor1 | Pmel | Sec24c | Tfcp2l1 | Ybx3 |
| Ap1b1 | Cep162 | Dnaic1 | Gnptg | Kpna6 | Necab3 | Pold1 | Sec31a | Thpo | Zbtb11 |
| Ap4m1 | Cep192 | Dnajb14 | Golga3 | Krit1 | Nek8 | Pole | Senp1 | Timm21 | Zbtb32 |
| Ap5z1 | Cep41 | Dnajc10 | Gon4l | Larp4 | Neu1 | Polr1a | Setd6 | Tjp2 | Zbtb48 |
| Apaf1 | Cers2 | Dnajc13 | Gpaa1 | Larp4b | Neurl4 | Polr2a | Setdb1 | Tlcd2 | Zdhhc12 |
| Apeh | Cfap20 | Dnajc14 | Gpatch2l | Lat2 | Nicn1 | Polr2k | Setdb2 | Tle4 | Zdhhc16 |
| Appl2 | Cfp | Dus2 | Grb10 | Lck | Ninl | Ppan | Sfi1 | Tle6 | Zfand2b |
| Araf | Chaf1a | Dync1li2 | Grina | Ldlr | Nipbl | Ppef2 | Sigirr | Tlk2 | Zfc3h1 |
| Arfgap1 | Chd1 | E2f5 | Gse1 | Lins | Nisch | Ppfia1 | Slc17a9 | Tmco6 | Zfp106 |
| Arfgef1 | Chd4 | Efcab7 | Gsr | Lipe | Nme4 | Ppfibp1 | Slc1a5 | Tmem147 | Zfp184 |
| Arhgap21 | Chd6 | Efemp2 | Gyg | Lonrf1 | Noc2l | Prkcg | Slc22a17 | Tmem165 | Zfp219 |
| Arhgef12 | Chd8 | Eif4g1 | Gys1 | Lrig2 | Noc3l | Prkd2 | Slc30a9 | Tmem19 | Zfp248 |
| Arid4a | Chfr | Ell3 | Haus5 | Lrmp | Noc4l | Prkra | Slc37a4 | Tmem199 | Zfp276 |
| As3mt | Cit | Elovl1 | Haus6 | Lrpprc | Nol12 | Prpf40b | Slc38a2 | Tmem87b | Zfp292 |
| Atat1 | Ckap5 | Eml2 | Hccs | Ltk | Nol7 | Prpf6 | Slc38a7 | Tmpo | Zfp335 |
| Atg4b | Ckmt1 | Enkd1 | Hcfc1 | Lztr1 | Nol8 | Prr11 | Slc39a7 | Tmub2 | Zfp385a |
| Atl3 | Clasrp | Eno2 | Hcfc2 | Macf1 | Notum | Prrc2a | Slc44a2 | Tnks | Zfp598 |
| Atp13a1 | Clcn2 | Eogt | Hdac4 | Man1b1 | Nphs1 | Prss36 | Slc44a4 | Tnpo2 | Zfp655 |
| Atp13a3 | Clcn6 | Ep300 | Heatr1 | Man2c1 | Npr1 | Psmb10 | Slc4a11 | Tns2 | Zgrf1 |
| Atp2a2 | Clic3 | Ep400 | Hectd1 | Map3k12 | Nprl2 | Psmc3ip | Slc50a1 | Top2b | Zmym5 |
| Atp2b4 | Clspn | Eps8l2 | Helq | Map4k3 | Nrbp1 | Ptpn6 | Slc52a2 | Top3b | Zp3 |
| Atr | Cnot1 | Eral1 | Helz | Map4k5 | Nsf | Ptprf | Slc7a6os | Topbp1 |  |
| Atrx | Cnot3 | Eri2 | Herc1 | Map7d2 | Nsmaf | Ptprv | Slco4c1 | Tpp2 |  |
| AU019823 | Cnot6 | Erlin2 | Herc2 | Mapk13 | Nsun5 | Pusl1 | Slfn9 | Trafd1 |  |
| AU041133 | Cntrob | Espl1 | Herpud2 | Marf1 | Nt5c | Rab24 | Smarce1 | Trappc8 |  |

1. Audic, S. and Claverie, J.M. (1997) The significance of digital gene expression profiles. *Genome Res*, **7**, 986-995.
