# Supplementary material for: IRFinder: assessing the impact of intron retention on mammalian gene expression
Source: Genome Biol. 2017 Mar 15;18:51. doi: 10.1186/s13059-017-1184-4 (PMC5353968; doi:10.1186/s13059-017-1184-4)
Supplement: Additional file 8: — Method used to detect enriched motifs associate with IR. (DOCX 50 kb) [file 13059_2017_1184_MOESM8_ESM.docx]

Motif analysis was performed by detecting enriched oligonucleotides relative to control datasets. As controls, 1000 introns that were least frequently retained were used. Subsequently, for each control, the enrichment of oligonucleotide sequences of length k, i.e. k-mers, was measured in two different ways: 1) Comparing the overall frequencies of k-mers in retained introns versus the frequencies from the control set, and 2) comparing the proportion of retained introns with a given k-mer versus the proportion of control introns with that k-mer. In both cases a z-score was calculated by comparing the observed value with the distribution of the control introns for each k-mer. Motif analysis of the 5' and 3' splice-sites are performed separately, to assess splice-site motifs independently of other possible splicing regulatory motifs.
