# Supplementary material for: IRFinder: assessing the impact of intron retention on mammalian gene expression
Source: Genome Biol. 2017 Mar 15;18:51. doi: 10.1186/s13059-017-1184-4 (PMC5353968; doi:10.1186/s13059-017-1184-4)
Supplement: Additional file 9: — Automatic detection of poly(A)-enriched samples. (DOCX 100 kb) [file 13059_2017_1184_MOESM9_ESM.docx]

# Automatic detection of polyA+ vs Ribo- samples

Table S8: Genes used to detect polyA+ vs Ribo- samples

| **non-polyadenylated genes used to detect Ribo- VS polyA+ library preparation** | | | | | | | | |
| --- | --- | --- | --- | --- | --- | --- | --- | --- |
| **SNORA80** | **SNORA71D** | **GPR21** | **HIST2H2AB** | **HIST1H2BI** | **SNORA40** | **SNORA34** | **HIST1H3F** | **HIST4H4** |
| **SNORA71C** | **AK311345** | **SNORD97** | **SNORA65** | **WDR38** | **HIST2H2AC** | **TVAS5** | **HIST1H4D** | **SNORA74B** |
| **SCARNA5** | **DCDC2B** | **HIST1H2AH** | **SCARNA21** | **SNORA48** | **SNORA81** | **SNORD12C** | **DQ597482** | **SNORD8** |
| **BC044606** | **SCARNA12** | **HIST1H2AM** | **SCARNA18** | **SNORA76** | **SCARNA6** | **HIST1H3E** | **HIST1H2BL** | **HIST1H2AK** |
| **HIST1H2BM** | **SNORA62** | **SNORD12B** | **HIST1H2BC** | **SCARNA1** | **HTR2B** | **HIST1H3J** | **HIST1H1C** | **HIST1H4I** |
| **HIST1H2AL** | **SNORA5A** | **HIST1H2AJ** | **AK096592** | **SNORA5C** | **SNORA37** | **HIST1H2AG** | **SNORA8** | **SNORA53** |
| **HIST1H1B** | **TAS2R19** | **HIST2H2BF** | **SCARNA9** | **ZNF124** | **SCARNA16** | **SNORA12** | **SCARNA9L** | **SNORA54** |
| **HIST1H4E** | **HIST1H2BF** | **SNORD17** | **BC079832** | **SNORA72** | **HIST1H2AB** | **SNORA57** | **SCARNA7** | **HIST1H3C** |
| **RPPH1** | **SCARNA10** | **SNORD60** | **C12orf61** | **CR605438** | **SNORA27** | **TMEM75** | **hCG_1818547** | **SCARNA2** |
| **SNORD18A** | **HIST1H4B** | **BC036695** | **HIST1H4A** | **TERC** | **LOC100129716** | **ZNF460** | **BC069756** | **HIST1H3B** |
| **CR590086** | **HIST1H2AC** | **BC070113** | **HIST2H2BE** | **SNORA23** | **SNORA3** | **SNORA63** | **HIST1H1E** | **SNORA84** |
| **SNORD10** | **SNORA18** | **VTRNA1-1** | **HIST1H3A** | **SNORA74A** | **HIST1H4C** | **ZNF678** | **AX748175** | **SNORA24** |
| **HIST1H2BG** | **SNORA68** | **HIST1H2BK** | **SNORD15A** | **SNORD2** | **HIST3H2A** | **HIST1H2BB** | **SNORD74** | **AX746867** |
| **SNORD94** | **SLC16A7** | **NAG8** | **SNORD46** | **HIST1H2AD** | **CR624170** | **SNORA52** | **SCARNA13** | **SCARNA4** |
| **SNORA67** | **HIST1H2BN** | **HIST1H4J** | **HIST1H3H** | **SNORD9** | **FLJ13224** | **SNORA21** | **C13orf25 v_1** |  |
| **RNU11** | **HIST1H3D** | **SNORA26** | **HIST1H2BO** | **SNORD55** | **DM004349** | **COL10A1** | **C8orf39** |  |
| **HIST1H3G** | **HIST1H2BH** | **HIST3H2BB** | **HIST1H4H** | **SNORA6** | **SNORA46** | **HIST1H2BJ** | **SNORA32** |  |
| **HIST1H2AE** | **SNORA14B** | **VTRNA1-3** | **BC021024** | **SNORA71B** | **RMRP** | **SNORA49** | **HIST1H2BD** |  |
| **SNORA13** | **HIST2H3D** | **SNORA31** | **SNORD15B** | **SCARNA20** | **SNORA20** | **SNORA28** | **DQ582265** |  |

Table S9: Samples used to validate polyA+ vs Ribo- classification

| PolyA+ | SRR357239 |
| --- | --- |
| PolyA+ | SRR522063 |
| PolyA+ | SRR522081 |
| PolyA+ | SRR522091 |
| PolyA+ | SRR522109 |
| PolyA+ | SRR522121 |
| PolyA+ | SRR900276 |
| PolyA+ | SRR900283 |
| PolyA+ | SRR900284 |
| PolyA+ | SRR900285 |
| PolyA+ | SRR900286 |
| PolyA+ | SRR900287 |
| PolyA+ | SRR900288 |
| PolyA+ | SRR900289 |
| PolyA+ | SRR900290 |
| Ribo- | SRR1027171 |
| Ribo- | SRR1027173 |
| Ribo- | SRR1027174 |
| Ribo- | SRR1027175 |
| Ribo- | SRR1027176 |
| Ribo- | SRR1027177 |
| Ribo- | SRR1027178 |
| Ribo- | SRR1027179 |
| Ribo- | SRR1027180 |
| Ribo- | SRR1027181 |
| Ribo- | SRR1027182 |
| Ribo- | SRR1027183 |
| Ribo- | SRR1027184 |
| Ribo- | SRR1027185 |
| Ribo- | SRR1027186 |


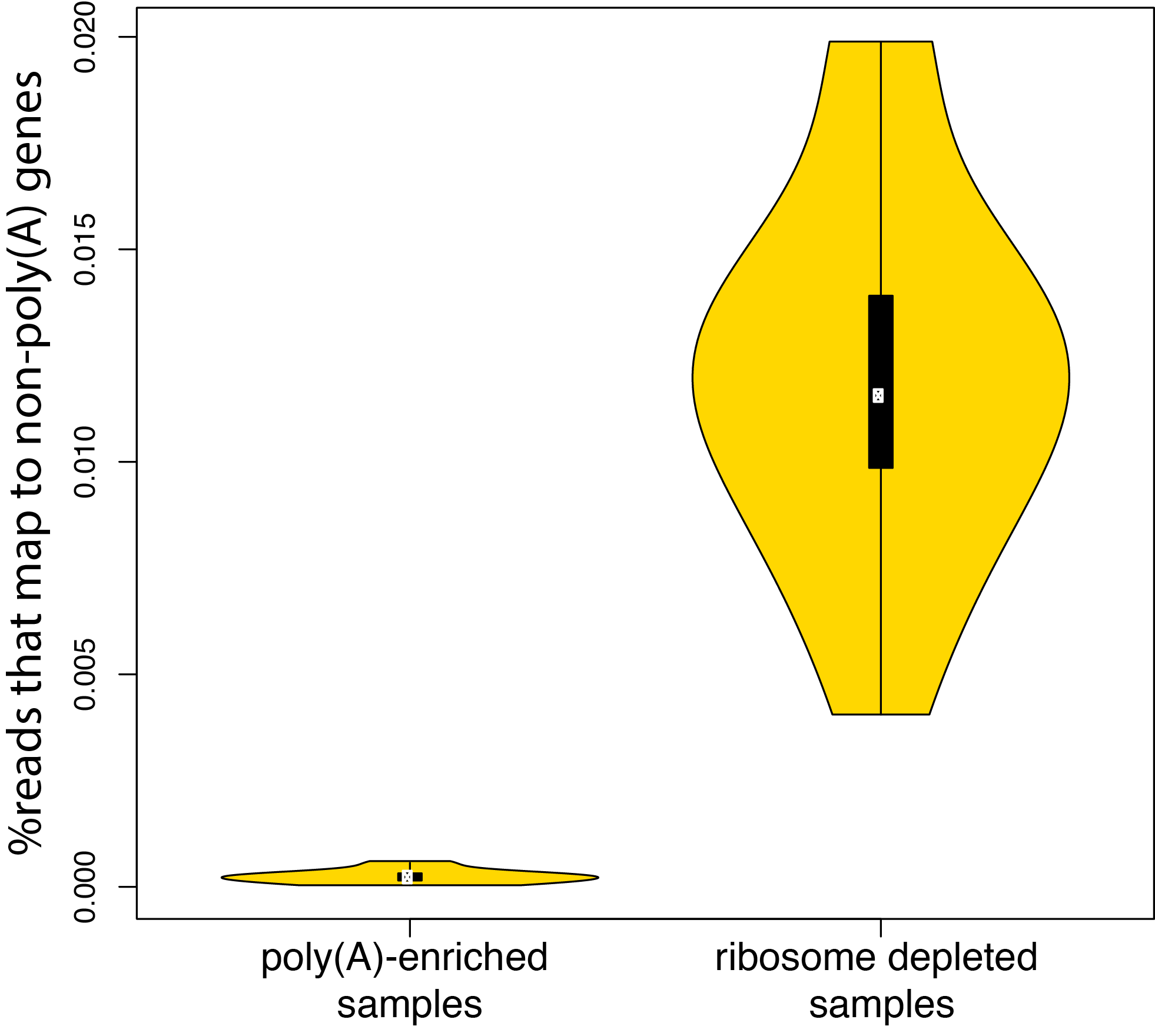


Figure S6: Number of reads that map to known non-poly(A) genes as a fraction of the total number of reads mapped to the main chromosomes (y-axis) for 15 libraries enriched for poly(A) (left) and 15 libraries that were only depleted of ribosomal RNA (right).
